# A Diastereoselective Synthesis of Dispiro[oxindole-cyclohexanone]pyrrolidines by 1,3-Dipolar Cycloaddition

**DOI:** 10.3390/molecules22122134

**Published:** 2017-12-04

**Authors:** Alexander Anis’kov, Irina Klochkova, Roman Tumskiy, Alevtina Yegorova

**Affiliations:** Institute of Chemistry, Chernyshevsky Saratov State University, 83 Astrakhanskaya Street, Saratov 410012, Russia; v-klochkov1@yandex.ru (I.K.); roma_ronaldinho@rambler.ru (R.T.); yegorovaay@gmail.com (A.Y.)

**Keywords:** spiro[indoline-3,2′-pyrrolidin]-2-one, azomethine ylides, unsaturated ketones, 1,3-dipolar cycloaddition, sarcosine

## Abstract

For the first time, arylmethylidene cyclohexanones that are non-symmetrical due to the presence of peripheral substituents were studied in 1,3-dipolar cycloaddition reactions. It is shown that the interaction with the azomethine ylide generated from sarcosine proceeds regio- and diastereoselectively, with the participation of two non-equivalent parts of the dipolarophile. Also for the first time, β-amino ketones (Mannich bases) were used as dipolarophile equivalents of unsaturated ketones. It was found that cycloaddition occurs diastereoselectively at the generated center.

## 1. Introduction

Recently, there great attention has been given in the literature to 1,3-dipolar cycloaddition reactions of azomethine ylides with alkenes activated by electron-withdrawing substituents. This is explained by the fact that azomethine ylides can be easily obtained from amino acids, benzylamine and isatin or ninhydrin [1,2,3]. Taking into account the large number of stereocenters formed during this process, the cycloaddition reaction proceeds with high stereoselectivity [3,4,5]. All this together makes the considered reactions a convenient tool for the construction of complex heterocyclic ensembles, structural analogues of natural alkaloids containing one or more *spiro*-atoms [5,6,7,8].

**Scheme 1:**
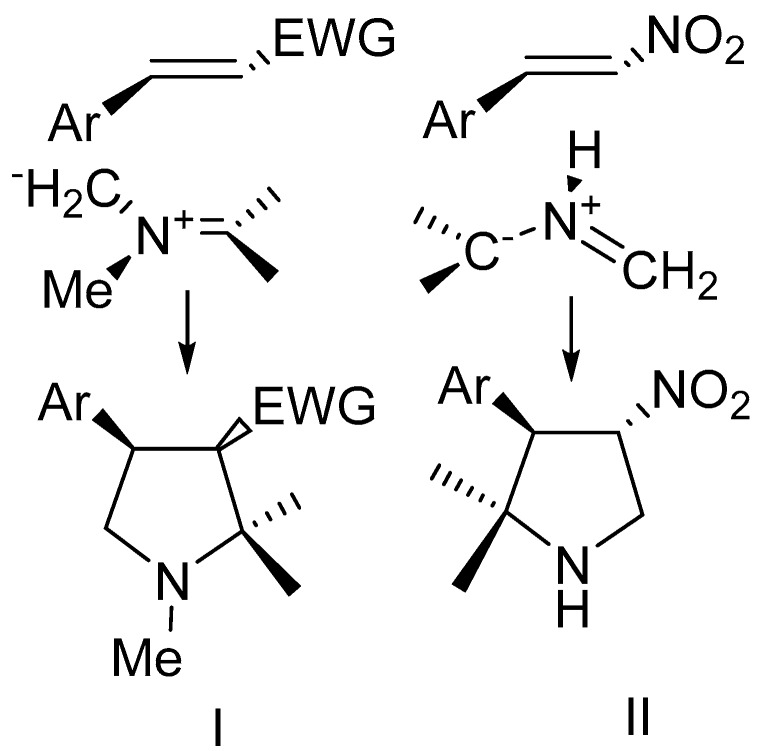
Cycloaddition of azomethine ylides.

Up to the present time, a large amount of data has been accumulated on the regio- and stereochemistry for dipolarophiles of different nature, including acrylates, chalcones, and nitrostyrenes, so it is known that ylides generated from sarcosine react regioselectively with the most electrophilic center of the dipolarophile [9,10,11,12,13] (path **I**, Scheme 1) with the formation of *endo*-adducts in good yields. Exceptions are nitrostyrene, that exhibits a reversed addition selectivity (path **II**, Scheme 1) [14]. The regioselectivity of interaction of dipoles generated from benzylamine and isatin with chalcones is less predictable [13,14,15]. Benzylamine ylide can form the addition product of the most electrophilic atom of the double bond to the less substituted carbon atom of the dipole (path I Scheme 1), but the opposite can also occur (path **II**, Scheme 1). Besides, the route depends on the reaction conditions and the acidity of the reaction medium [15].

However, all these data relate to activated alkenes having one reaction center, and there is much less data on the use of dipolarophiles with two non-equivalent and reactive centers. The use of such dipolarophiles raises the question of selectivity of addition, the solution of which can provide valuable information on the details of the course of the reaction.

Previously, cycloaddition of sarcosine ylide to non-symmetrical (due to the presence of peripheral substituents) diarylmethylideneacetones was studied [12]. It was shown that the addition of ylide occurs at the most electrophilic center of the asymmetric dienone. However, the reactions of non-symmetrical biarylmethylidenecyclohexanones [16], as more sterically hindered analogues, with sarcosine and benzylamine ylides have not been studied. Therefore, it was interesting to evaluate the activity of the non-equivalent centers and the regio- and stereoselectivity of cycloaddition of azomethine ylides to such sterically hindered substrates. Previously, these ketones were systematically studied in reactions with nucleophiles (hydrazine and phenylhydrazine) [16]. Differences in the activity of the multiple bonds of the substrates were established.

## 2. Results and Discussion

**Scheme 2:**
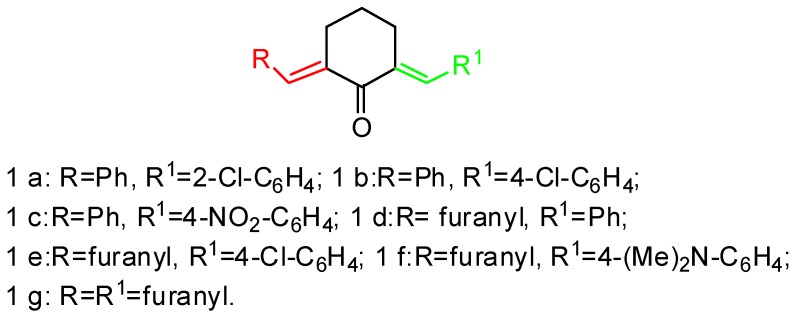
Initial substrates.

We used diarylmethylidenecyclohexanones as initial substrates, **1a**–**g** (Scheme 2), which differ in the peripheral substituents.

**Scheme 3:**
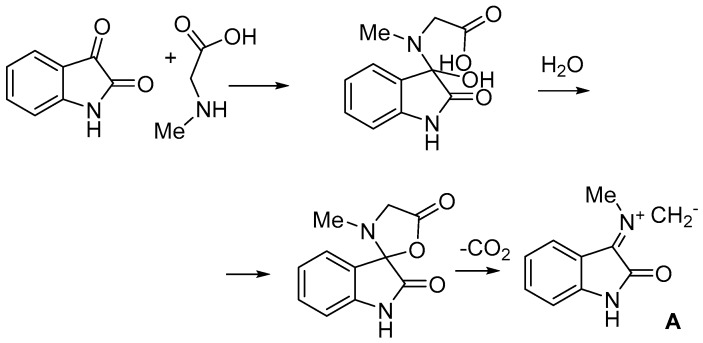
Generation of azomethine ylide **A**.

As model dipoles, we selected azomethine ylide **A** generated from sarcosine and isatin, as the most accessible in terms of their generation (Scheme 3).

**Scheme 4:**
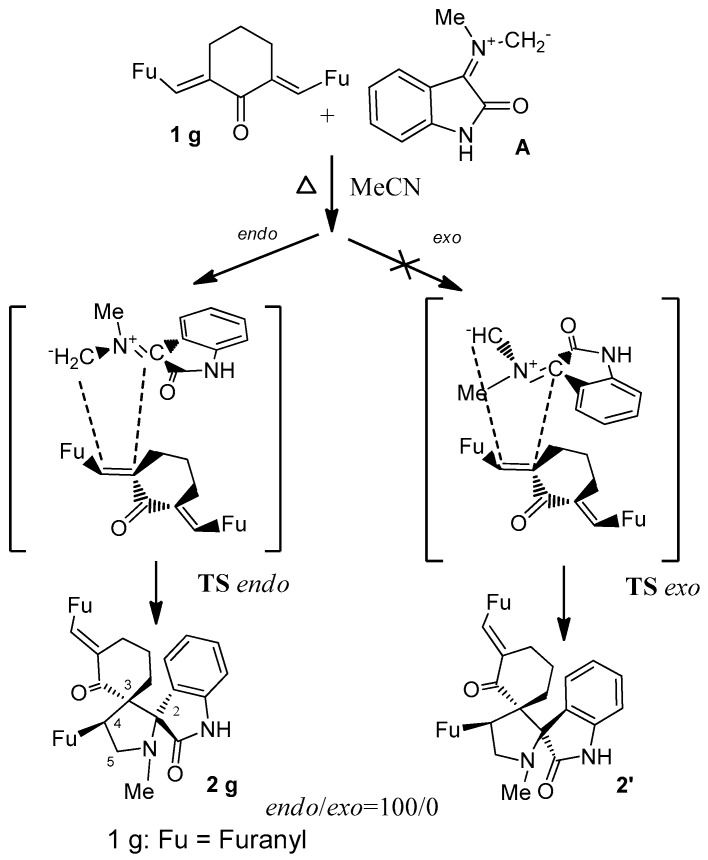
Cycloaddition of ylide **A** to difurylmethylidencyclohaxanone **1g**.

The reaction was carried out as a three component interaction of equimolar amounts of ketone **1**, isatin and sarcosine by refluxing in acetonitrile for 3–5 h. The same solvent was used by us in similar studies performed earlier [12]. 1,3-Dipolar cycloaddition is a highly stereoselective reaction. The stereoselective formation of an *endo*- or an *exo*-adduct can be realized in the result of the cycloaddition. Sometimes, the reaction leads to the formation of a mixture thereof. The probability of favoring one or another direction is determined by a combination of factors. The substrates **1a**–**e** contain two unequal parts of the dipolarophile (green or red on Scheme 2), which raises the question about the direction of attack of the dipole. However, in view of the question of *endo* or *exo*-stereoselectivity, the identification of products may become more complicated. It can be easily solved by using a dipolarophile whose structure does not contain a second non-equivalent center. Despite the existence of literature data concerning the use of such dipolarophiles [17], we have performed a control experiment for the selected solvent and we used difurylmethylidenecyclohexanone **1g** as an initial compound (Scheme 4).

**Figure 1:**
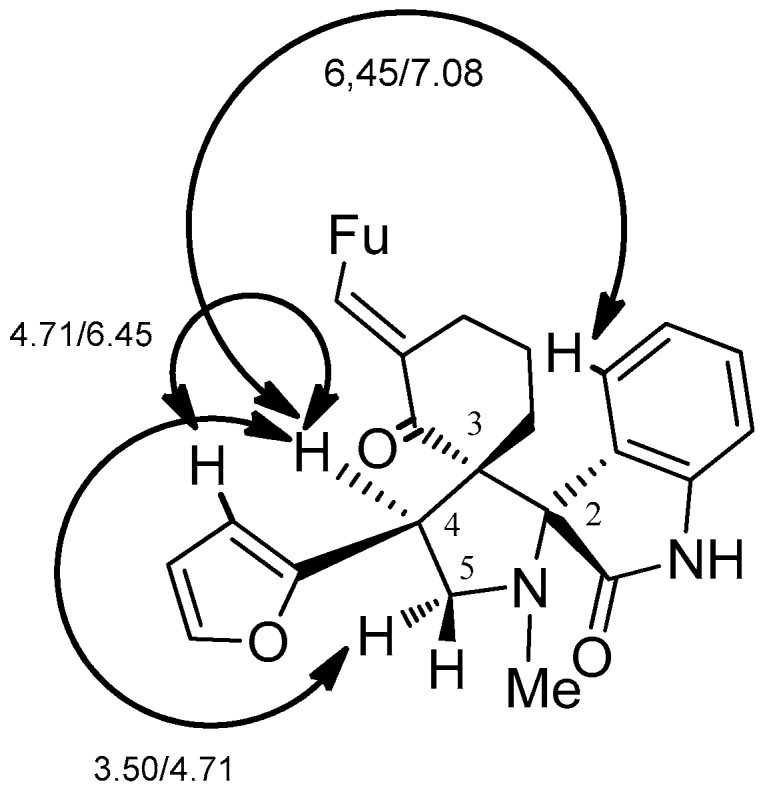
The key NOE contacts for **2g**.

Based on the combination of TLC and NMR spectroscopy data, we established that the cycloaddition of the sarcosine ylide **A** with **1g** takes place regio- and diastereoselectively to give *endo*-adduct **2g**. The ^1^H-NMR spectra of **2g** have three characteristic triplets at 3.50 ppm (t, *J* = 8.6 Hz, 1H), at 3.85 ppm (t, *J* = 9.5 Hz, 1H) and 4.71 ppm (t, *J* = 9.1 Hz, 1H), respectively. This signals are related to the AMX system of protons H_2_-5 and H-4 of pyrrolidine ring. In the ^13^C-NMR spectrum, the signals corresponding to C-4 and C-5 were observed at 43.2 ppm, 57.2 ppm. The quaternary carbons C-2 and C-3 were resonated at 62.2 ppm, and 72.3 ppm, respectively. These signals indicate that pyrrolidine ring is connected with the oxindole and cyclohexanone moieties by two *spiro*-carbons. The relative configuration of the stereogenic centers has been established on the basis of the NOESY data (see Supplementary Materials). The key cross correlations are shown in Figure 1. The diagnostic strong correlation of the aromatic proton of oxindole and H-4 of pyrrolidine ring confirmed the stereochemistry of stereogenic centers.

**Scheme 5:**
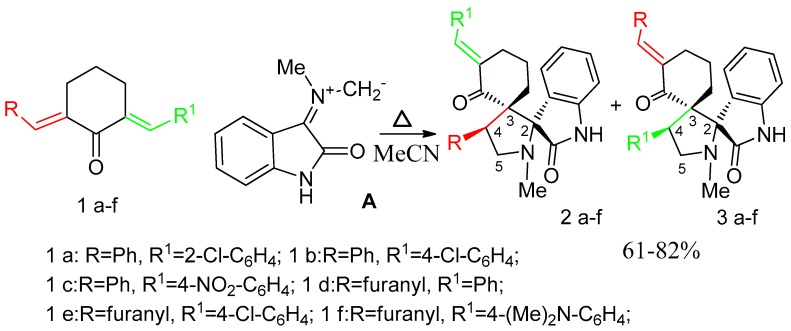
Cycloaddition of ylide **A** to diarylmethylidenecyclohexanones **1a**–**f**.

The formation of the diastereomer **2g** occurs through the *endo*-transition state (**TS** *endo*), as the most advantageous cycloaddition channel. The *exo*-channel (**TS** *exo*) is not beneficial, because of the repulsion of the cyclohexane core and the oxindole fragment. The results of our control experiment are confirmed by literature data [9,10,11]. Under similar conditions, biarylidenecyclohexanones **1a**–**f** (Scheme 5) in the reaction with sarcosine ylide give rise to a mixture of **2** and **3** cycloaddition products at two reactive multiple bonds.

The ^1^H-NMR spectra exhibited two triplets of diastereotopic protons H_2_-5 at 3.39–3.83 ppm, 3.49–3.99 ppm (*J* = 8.8–9.1 Hz). Two doublet of doublets or multiplets at 4.73–4.86, 4.81–5.17 ppm (dd, *J* = 10.6, 7.6 Hz) were assigned to H-4 of pyrrolidine ring of compounds **2** and **3**, respectively. For some compounds (**2** and **3b**) a coalescence of signals of protons at the C-5 and C-4 positions is observed. The chemical shift of protons at C-4 isomers **3** depends on the nature and the position of substituent in the ring. Proton H-4 in **3a** was resonated at 5.17 ppm in the form of a triplet. It is due to the influence of the chlorine atom in the *ortho*-position of the ring. The signal at similar position in **3c** is weakly deshielded. It was observed at 4.95 ppm, which is due to the influence of the nitro group. The signals of the considered position for **2a**, **c**, **3d** and **2d**–**f** were observed in the interval 4.81–4.86 ppm and 4.70–4.74 ppm, respectively.

In the ^13^C-NMR spectrum the peaks at 47.7–49.3 ppm, and 60.3–60.9 ppm were assigned to pyrrolidine carbons C-4 and C-5, respectively. The two *spiro*-carbons resonate at 63.8–64.2 ppm, and 76.4–76.8 ppm. The carbonyl carbon of oxindole fragment was noted at 171.2–177.2 ppm. Also carbons of carbonyl group of cyclohexane skeleton was observed at 200.1–201.2 ppm. In most cases, a doubling of the signals were observed in the ^13^C-NMR spectra, which confirms the presence of two isomers with similar structures. Unfortunately, the preparative separation of the isomers of **2** and **3** were hindered by their similar chromatographic mobility.

**Figure 2:**
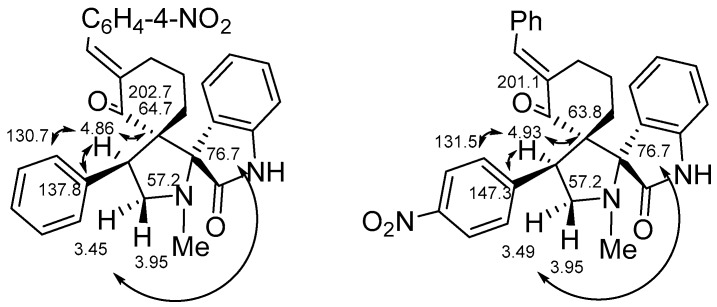
The key HMBC correlation for **2** and **3c**.

The exact structural identification of compounds **2** and **3** were carried out on the basis of the ^1^H^13^C HMBC experiments (see Supplementary Materials). For example, in the spectra of products **2** and **3c** important cross-peaks, proving the formation of **2c**, were noted (Figure 2): 4.86/137.8 ppm, 4.86/130.7 ppm. That cross-peaks correspond to correlations of the proton at C-4 of pyrrolidine fragment with *ipso*- and *ortho*-carbons of the benzene ring. The same correlations were noted for **3c**: 4.93/141.3 ppm, 4.93/131.5 ppm (Figure 2). The correlations of protons at C-4 of the pyrrolidine fragment with *spiro*-carbons (4.86/64.7 ppm, 4.86/76.7 ppm), (4.93/63.8 ppm, 4.86/76.7 ppm), for **2** and **3c**, respectively were observed.

**Figure 3:**
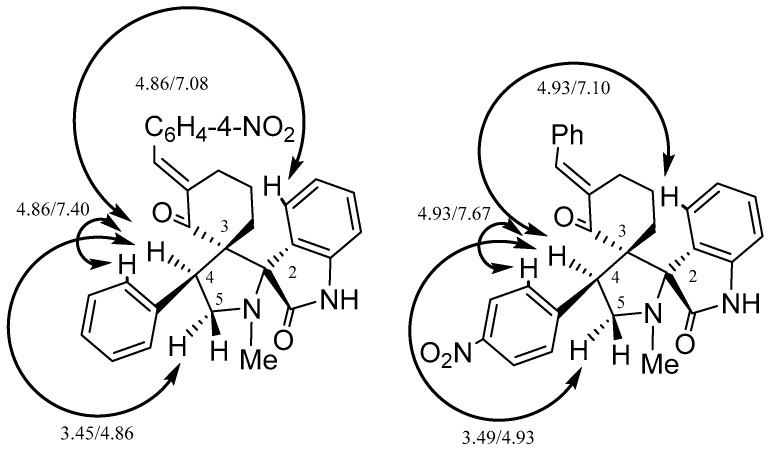
The key NOE contacts for **2** and **3c**.

Moreover, the structures of the adducts **2** and **3c** were confirmed by the data of NOESY spectroscopy (see Supplementary Materials). For example, key NOE-contacts between protons at C-4 with *ortho*-hydrogens of aromatic rings (4.86/7.40 ppm for **2c** and 4.93/7.67 ppm for **3c**) were noted. (Figure 3). On the basis of NOESY data the *endo*-configuration of adduct was discovered. Also, the key correlations between protons at C-4 with proton of the oxindole fragment for **2** and **3c** (4.86/7.08 ppm and 4.93/7.10 ppm for **2** and **3c**, respectively) were observed. The same configuration of compounds **2d**–**f** and **2d** was confirmed by close chemical shifts and the coupling constants of the protons at C-4.

Guided by the analysis of the relative intensities of key signals (CH protons of the pyrrolidine fragment) in ^1^H-NMR spectra, we established the ratio of isomers **2** and **3** (Table 1). Thus, in the case of ketones **1d**–**f** the furylmethylidene moiety is a strongly dominant center. These facts can be explained by the greater steric availability of the furylmethylidene fragment (red part in Scheme 2) of dipolarophiles **1d**–**f** for attack of azomethine ylide. There were not dominated centers of cycloadition for dipolarophiles **1a**–**c**. The quantities of isomers **2** and **3a**–**c** were the same. It should be noted that the *ortho*-substituent in the ring makes the cycloaddition less preferable.

It was discovered that the diastereoselectivity does not depend on the nature of the substituent at the reaction centers of the dipolarophile. All products were *endo*-adducts. Thus, the most electrophilic and sterically available center of the dipolarophile reacts with the more accessible carbon of the dipole. Steric repulsion of the cyclohexane skeleton and oxindole ring makes the *exo*-transition state (**TS** *exo*) not fruitful.

**Scheme 6:**
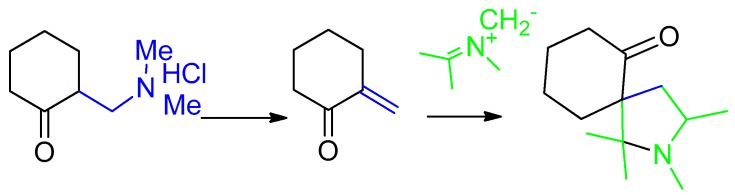
Cycloaddition of azomethine ylides to the Mannich ketones.

Next we studied the chemical behavior of Mannich ketones in reactions with sarcosine ylide. We proposed that Mannich bases can react with azomethine ylide **A**, because they can generate unsubstituted unsaturated ketones at the β-position (Scheme 6). We also proposed, that they can form an unsubstituted pyrrolidines at the 4 or 5 positions.

**Scheme 7:**
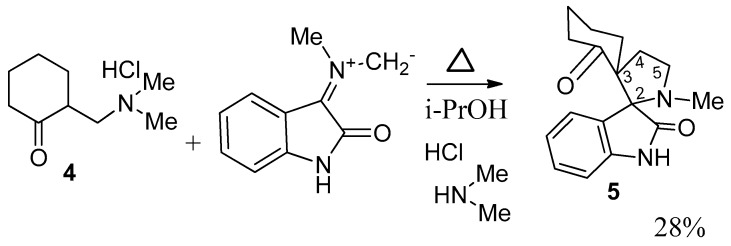
Cycloaddition of ylide **A** to the unsaturated ketone generated from hydrochloride 2-[(dimethylamino)methyl]cyclohexanone.

It is known, that cyclohexanone Mannich bases are the simplest in deamination reaction [18]. We used 2-[(dimethylamino)methyl]cyclohexanone hydrochloride (**4**) (Scheme 7) as the simplest dipolarophile. We don’t use acetone derivatives as the most simple dipolarophile due to the bad results [18]. 2-[(dimethyl-amino)methyl]cyclohexanone hydrochloride (**4**) reacted with azomethine ylide **A** with the formation of dispirane **5** in 28% yield. The reaction was carried out as a three component interaction of equimolar amounts of ketone **4**, isatin and sarcosine by mixing in isopropanol at 60 °C for 3–5 h. Refluxing in isopropanol led to very low yield of **5**. When acetonitrile was used as a solvent, the recovery of the reaction product was greatly complicated by the formation of a resin.

The ^1^H-NMR spectrum of the synthesized compound **5** exhibits multiplet at 3.20–3.28 ppm, which correspond to proton at C-5 of pyrrolidine ring. Also two doublet of doublet of doublets at 2.66 ppm (*J* = 10.7, 8.5, 5.4 Hz), and at 1.93 ppm (*J* = 10.8, 8.3, 4.2 Hz) were noted. These signals were assigned to two diastereotopic protons at C-4 of pyrrolidine ring.

In the ^13^C-NMR spectrum, 34.1 ppm, 52.1 ppm signals were assigned to the С-4 and C-5 of pyrrolidine ring. The signals of quaternary carbons, which were resonated at 62.1 ppm, and 76.1 ppm, were noted. This was evidence of the formation of a new heterocyclic system, where the pyrrolylidine ring is connected to the oxindole and cyclohexanone skeletons through two *spiro* carbons.

**Scheme 8:**
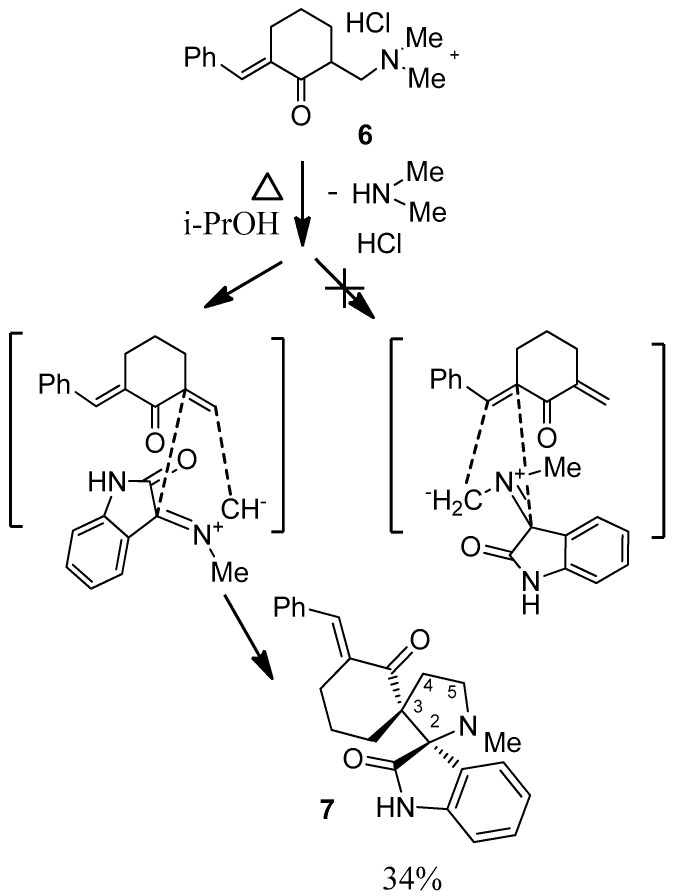
Cycloaddition of ylide **A** to the unsaturated ketone generated from **7**.

We decided to continue the research. The structure of the initial dipolarophile was complicated by the introduction of a new reaction center (phenylmethylidene fragment). **6**. The introduction of that center will allow studying the selectivity of cycloaddition. It is established that sarcosine ylide reacts with dipolarophile **6** regioselectively and generates spirane **7** (Scheme 8).

In the ^1^H-NMR spectrum of dispirane **7** signals of diastereotopic protons at C-5 of the pyrrolidine ring at 3.49 ppm ddd (*J* = 9.3, 5.6, 3.6 Hz) and 3.25 ppm ddd (*J* = 11.5, 5.6, 2.2 Hz) were noted. The hydrogen of the ylidene fragment at 7.42 ppm was observed. This fact proved the course of cycloaddition with the participation of only the aminomethylene center. It was difficult to identify of the signals of protons at C-4 due to coalescence. The chemical shifts of diastereotopic protons at C-4 (2.51 and 2.17 ppm) were determined using the dqCOSY experiment. The observation of the two signals of *spiro* carbons at 59.9 ppm, 78.3 ppm in ^13^C-NMR spectrum of **7** confirms the formation of new pyrrolidine cycle. The signals of two carbonyl carbons at 178.9 ppm, 212.3 ppm were noted.

**Figure 4:**
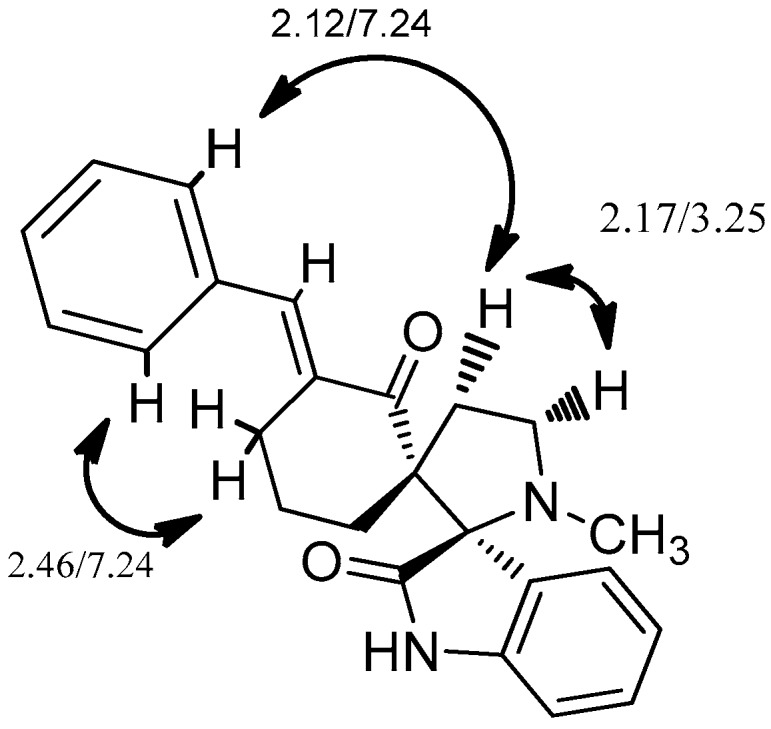
The key NOE contacts for **7**.

Based on the NOESY 2D experiment data, we have established the spatial architecture of the molecule (Figure 4). Key NOE contacts are marked on the diagram. Adduct **7** is the result of cycloaddition of ylide **A** to the most sterically available center (see Supplementary Materials). These data correlate well with data on the selectivity of cycloaddition to dipolarophiles **1a**–**f**.

Arylmethylidene cyclohexanones that are on-symmetrical due to peripheral substituents were studied in the reactions of 1,3-dipolar cycloaddition for the first time. It was shown that two non-equivalent centers of the dipolarophile react with azomethine ylide **A**. The direction of cycloaddition depends on the steric volume of the peripheral substituent at **1a**–**f**. It was discovered that the most sterically available and electrophilic center of the dipolarophile reacts with the more accessible carbon of the ylide. This is also confirmed by the data of the reactions of Mannich ketones. Cycloaddition took place only at the aminomethylene center.

## 3. Materials and Methods

The ^1^H- and ^13^C-NMR spectra were recorded at 25 °C on a Varian-400 spectrometer (400 and 100 MHz, respectively; Agilent Technologies, Santa Clara, CA, USA), using CDCl_3_ as a solvent and tetramethylsilane as an internal standard. Analytical thin layer chromatography TLC was performed using Alugram Xtra Sil G 254 plates (Macherey-Nagel GmbH & Co. KG, Düren, Germany; hexane–ethyl acetate–chloroform, 2:2:1). The melting points were measured in open capillaries. The elemental analyses were obtained on a Vario Micro cube Elementar CHNS analyzer (Elementar Analysensysteme GmbH, Hanau, Germany). Arylmethylidenecyclohexanones were synthesized according to [16]. Diarylmethylidenecyclohexanones **1a**–**f** were synthesized according to [19]. 2-((Dimethyl)methyl)cyclohexanone hydrochloride (**4**) and 6-phenylmethylidene-2-((dimethyl)methyl)-cyclohexanone hydrochloride (**6**) were synthesized according to [20,21].

### 3.1. Synthesis of 1-N-Methyl-spiro[2.3^1^]oxindole-spiro[3.2^11^]6^11^-arylmethylidenecyclohexanone-4-aryl-pyrrolidines (General Method). ***2**, **3a**–**g***

A solution of ketone **1a**–**f** (5 mmol), isatin (0.88 g, 6 mmol), and sarcosine (0.45 g, 5 mmol) in acetonitrile (20 mL) was heated at reflux for 3–5 h. The solvent was evaporated off at reduced pressure, and the solid residue was washed with hexane.

*1-N-Methylspiro[2.3^1^]oxindole-spiro[3.2^11^]6^11^-(2-chlorophenyl)methylidenecyclohexanone-4-phenyl-pyrrolidines and 1-N-methylspiro[2.3^1^]oxindole-spiro[3.2^11^]6^11^-phenylmethylidenecyclohexanone-4-(2-chloro-phenyl)pyrrolidines* (**2**, **3a**). Yield: 78%. m.p.: 171–173 °C. ^1^H-NMR: δ 1.02–1.19 (m, 1H), 1.21–1.42 (m, 1H), 2.09 (c, 1.08H), 2.11 (c, 1.92H), 2.16–2.46 (m, 4H), 3.44 (t, *J* = 8.3 Hz, 0.64H, C-5), 3.49 (t, *J* = 8.9 Hz, 0.36H, C-5), 3.91 (t, *J* = 8.9 Hz 0.64H, C-5), 3.99 (t, *J* = 8.9 Hz, 0.36H, C-5), 4.86 (dd, *J* = 11.9 Hz, 8.0 Hz 0.64H, C-4), 5.17 (t, *J* = 8.9 Hz 0,36Н, C-4), 6.70–6.80 (m, 1H), 6.90 (d, *J* = 7.4 Hz, 1H), 7.01 (t, *J* = 7.8 Hz, 1H), 7.62–7.14 (m, 9H), 8.00 (d, *J* = 7.9 Hz, 1H), 8.05 (broad s, 0.36H), 8.07 (broad s, 0.64H). ^13^C-NMR: δ 28.3, 28.4, 30.9, 31.2, 34.7, 34.9 (Me), 37.7, 45.6, 47.7 (C-4), 49.4 (C-4), 52.8, 57.5 (C-5), 57.7 (C-5), 62.2 (spiro), 63.8 (spiro), 76.1 (spiro), 109.3, 109.4, 123.5, 126.6, 126.8, 127.8, 128.1, 128.2, 128.2, 128.5, 128.7, 129.1, 129.2, 129.3, 129.4, 129.9, 130.2, 130.4, 134.3, 134.6, 135.3, 138.5, 139.3, 141.2, 171.2, 177.2, 201.3 (C=O), 201.6 (C=O). Anal. Calcd. for C_30_H_27_ClN_2_O_3_: C 74.60, H 5.63, N 6.63; found: C 75.01, H 5.54, N 6.96.

*1-N-Methylspiro[2.3^1^]oxindole-spiro[3.2^11^]6^11^-(4-chlorophenyl)methylidenecyclohexanone-4-phenyl-pyrrolidines and 1-N-methylspiro[2.3^1^]oxindole-spiro[3.2^11^]6^11^-phenylmethylidenecyclohexanone-4-(4-chloro-phenyl)pyrrolidines* (**2**, **3b**). Yield: 72%. m.p.: 175–178 °C. 3,4 b ^1^H-NMR: δ 1.03–1.16 (m, 1H), 1.23–1.66 (m, 1H), 2.11 (с, 1.5H), 2.12 (c, 1.5 H), 2.15–2.25 (m, 2H), 2.30–2.47 (m, 2H), 3.43 (t, *J* = 8.9 Hz, 1H, C-5), 3.83 (t, *J* = 8.9 Hz, 0.5H, C-5), 3.90–3.95 (m, *J* = 8.9 Hz, 0.5H, C-5), 4.87–4.78 (m, 1H, C-4), 6.72 (d, *J* = 7.7 Hz, 1H), 6.90–7.10 (m, 2H), 7.06–7.27 (m, 9H), 7.34–7.16 (m, 6H), 7.80 (broad s, 1H). ^13^C-NMR: δ 21.3, 28.5, 30.9, 31.0, 34.7(Me), 48.7 (C-4), 49.3(C-4), 57.8 (C-5), 63.8 (spiro), 64.0 (spiro), 76.6 (spiro), 109.3, 122.6, 122.7, 126.8, 128.0, 128.2, 128.4, 128.3, 128.4, 129.8, 130.3, 131.0, 134.1, 134.2, 136.9, 152.9, 153.1, 171.2, 202.3 (C=O). Anal. Calcd. for C_30_H_27_ClN_2_O_3_: C 74.60, H 5.63, N 6.63; found: C 74.52, H 5.33, N 6.55.

*1-N-Methylspiro[2.3^1^]oxindole-spiro[3.2^11^]6^11^-(4-nitrophenyl)methylidenecyclohexanone-4-phenyl-pyrrolidines and 1-N-methylspiro[2.3^1^]oxindole-spiro[3.2^11^]6^11^-phenylmethylidenecyclohexanone-4-(4-nitro-phenyl)pyrrolidines* (**2**, **3c**). Yield: 82%. m.p.: 191–193 °C. ^1^H-NMR: δ 1.00–1.10 (m, 1H), 1.11–1.18 (m, 1H), 1.26–1.44 (m, 2H), 2.12 (s, 1.35H), 2.14 (s, 1.65H), 2.19–2.47 (m, 2H), 3.38–3.51 (m, 1H), 3.92 (dd, *J* = 10.6, 7.7 Hz, 0.55H, C-4), 4.86 (dd, *J* = 10.6, 7.6 Hz, 0.45H, C-4), 4.95 (dd, *J* = 10.1, 7.7 Hz, 0.55H), 6.75 (d, *J* = 7.7 Hz, 1H), 7.01–7.12 (m, 2H), 7.13–7.36 (m, 5H), 7.44 (d, *J* = 7.3 Hz, 1.08H), 7.66 (d, *J* = 8.6 Hz, 0.92H), 7.80 (broad s, 1H), 8.12 (d, *J* = 8.7 Hz, 0.92H), 8.17 (d, *J* = 8.7 Hz, 1.08H). ^13^C-NMR: δ 21.4, 28.7, 30.7, 31.8, 34.5 (Me), 48.8 (C-4), 49.0 (C-4), 57.2 (C-5), 63.8 (spiro), 64.7 (spiro), 76.7 (spiro), 109.3, 122.4, 122.6, 122.9, 126.5, 128.1, 128.2, 128.3, 128.3, 128.5, 129.8, 130.3, 131.0, 136.1, 135.2, 136.8, 141.1, 141.2, 146.8, 147.3, 171.2, 201.7 (C=O), 202.1 (C=O). Anal. Calcd. for C_30_H_27_N_3_O_4_: C 73.01, H 5.51, N 8.51; found: C 73.38, H 5.37, N 8.56.

*1-N-Methylspiro[2.3^1^]oxindole-spiro[3.2^11^]6^11^-2-furylmethylidenecyclohexanone-4-phenyl-pyrrolidines and 1-N-methylspiro[2.3^1^]oxindole-spiro[3.2^11^]6^11^-phenylmethylidenecyclohexanone-4-2-furylpyrrolidines* (**2**, **3d**). Yield: 65%. m.p.: 164–168 °C. ^1^H-NMR: δ 1.14–1.25 (m, 2H), 1.24–1.44 (m, 2H), 2.13 (s, 3H), 2.23–2.49 (m, 2H), 3.45 (t, *J* = 7.7 Hz, 1H), 3.83 (t, *J* = 7.9 Hz, 0.2H), 3.91 (t, *J* = 7.9 Hz, 0.8H), 4.74 (dd, *J* = 10.1, 7.7 Hz, 0.2H), 4.81 (dd, *J* = 10.1, 7.7 Hz, 0.8H), 6.40 (d, *J* = 3.2 Hz, 1H), 6.45 (d, *J* = 3.3 Hz, 1H), 6.73 (d, *J* = 7.6 Hz, 1H), 6.91 (t, *J* = 7.6 Hz, 1H), 7.07–7.15 (m, 2H), 7.17–7.34 (m, 5H), 7.43 (d, *J* = 1.5 Hz, 1H), 7.47 (d, *J* = 7.5 Hz, 2H), 7.93 (broad s, 1H). ^13^C-NMR: δ 22.8, 27.9, 28.5, 30.5, 34.7, 49.4 (C-4), 55.9 (C-5), 58.1 (C-5), 62.8, 68.4 (spiro), 76.7 (spiro), 108.4, 109.2, 112.2, 115.7, 122.8, 125.4, 126.7, 128.11, 128.4, 129.2, 130.6, 138.4, 139.4, 141.3, 144.4, 152.5, 177.2, 201.3 (C=O).

*1-N-Methylspiro[2.3^1^]oxindole-spiro[3.2^11^]6^11^-2-furylmethylidenecyclohexanone-4-4-chlorophenyl-pyrrolidines and 1-N-methylspiro[2.3^1^]oxindole-spiro[3.2^11^]6^11^-4-chlorophenylmethylidenecyclohexanone-4-2-furylpyrrolidines* (**2**, **3e**). Yield: 68%. m.p.: 164–168 ^1^H-NMR: δ 1.23–1.48 (m, 2H), 1.80–1.93 (m, 1H), 2.05 (s, 3H), 2.18–2.38 (m, 1H), 2.39–2.56 (m, 1H), 3.53–3.36 (m, 1H), 3.83 (t, *J* = 9.6 Hz, 1H), 4.72 (dd, *J* = 10.1, 7.6 Hz, 0.8H), 4.79 (dd, *J* = 10.1, 7.5 Hz, 0.2H), 6.40 (d, *J* = 3.2 Hz, 1H), 6.45 (d, *J* = 3.3 Hz, 1H), 6.36–6.52 (m, 2H), 6.64 (d, *J* = 3.2 Hz, 1H), 6.72 (t, *J* = 7.3 Hz, 1H), 6.90 (t, *J* = 7.8 Hz, 1H), 7.03–7.18 (m, 2H), 7.20–7.35 (m, 1H), 7.43 (d, *J* = 7.6Hz, 2H), 7.53 (d, *J* = 1.5 Hz, 1H), 8.02 (broad s, 1H). ^13^C-NMR: δ 18.2, 18.3, 19.2, 21.7, 25.4, 27.9, 27.9, 29.1, 30.6, 34.6, 34.7, 43.3, 43.6 (C-4), 49.3 (C-4), 55.9 (C-5), 56.3 (C-5), 62.5 (spiro), 64.5 (spiro), 77.5 (spiro), 108.4, 108.5, 109.3, 110.3, 112.3, 115.7, 115.9, 116.1, 122.8, 123.4, 125.6, 128.2, 128.4, 128.4, 129.3, 131.9, 132.6, 138.0, 141.3, 144.5, 152.5, 152.6, 152.8, 154.4, 177.2, 200.7 (C=O), 201.2 (C=O). Anal. Calcd. for C_28_H_25_ClN_2_O_3_: C 71.10, H 5.33, N 5.92; found: C 70.95, H 4.97, N 5.77.

*1-N-Methylspiro[2.3^1^]oxindole-spiro[3.2^11^]6^11^-2-furylmethylidenecyclohexanone-4-4-N,N-(dimethyl)phenyl-pyrrolidines and 1-N-methylspiro[2.3^1^]oxindole-spiro[3.2^11^]6^11^-4-N,N-(dimethyl)phenylmethylidenecyclo-hexanone-4-2-furylpyrrolidines* (**2**, **3f**) Yield: 64%. m.p.: 131–134 ^1^H-NMR: δ 1.23–1.51 (m, 2H), 1.80–2.10 (m, 5H), 2.39–2.49 (m, 2H), 2.91 (c, 0.9H), 3.09 (c, 2.1H), 3.49 (t, *J* = 8.6 Hz, 1H), 3.80–3.87 (m, 1H), 4.68 (t, *J* = 8.6, 0.3H), 4.70 (dd, *J* = 8.5 Hz, 0.7H), 6.34 (d, *J* = 3.2 Hz, 1H), 6.41–6.47 (m, 1H), 6.47–6.52 (m, 1H), 6.63–6.70 (m, 1H), 6.72 (d, *J* = 7.3 Hz, 1H), 6.89–6.92 (m, 2H), 7.06 (d, *J* = 7.3 Hz, 1H), 7.12 (t, *J* = 7.3 Hz, 1H), 7.32 (d, *J* = 7.6Hz, 2H), 7.47 (d, *J* = 1.5 Hz, 0.7H), 7.55 (d, *J* = 1.5 Hz, 0.3H), 8.03 (broad s, 1H). ^13^C-NMR: δ 18.2, 19.8, 21.7, 25.4, 28.0, 29.1, 34.7, 40.7, 40.9, 43.6, (C-4), 56.3 (C-5), 62.2 (spiro), 77.5 (spiro), 108.5, 109.3, 110.4, 112.4, 112.6, 115.8, 115.9, 122.2, 125.3, 125.7, 128.2, 128.6, 128.9, 129.3, 132.8, 141.2, 144.55, 152.6, 152.8, 154.4, 173.9, 200.8 (C=O). Anal. Calcd. for C_30_H_31_N_3_O_3_: C 74.82, H 6.49, N 8.73; found: C 74.52, H 6.54, N 8.62.

*1-N-Methylspiro[2.3^1^]oxindole-spiro[3.2^11^]6^11^-2-furylmethylidenecyclohexanone-4-2-furylpyrrolidines* (**3g**) Yield: 61%. m.p.: 163–165 ^1^H-NMR: δ 1.23–1.59 (m, 4H), 2.13 (s, H), 2.41–2,50 (m, 2H), 3.50 (t, *J* = 8.6 Hz, 1H), 3.85 (t, *J* = 9.5 Hz, 1H), 4.71 (t, *J* = 9.1 Hz, 1H), 6.35 (dd, 1H, *J* = 3.5, 1.8 Hz, 1H), 6.45 (d, *J* = 4.4 Hz, 1H), 6.50 (d, *J* = 4.4 Hz, 1H),6.73 (d, *J* = 7.7 Hz, 1H), 6.90 (t, *J* = 7.6 Hz, 1H), 7.08 (d, 1H, *J* = 7.7), 7.12 (t, 1H, *J* = 7.5), 7.33 (d, *J* = 4.8 Hz, 2H), 7.47 (s, 1H), 7.85 (broad s, 1H). ^13^C-NMR: δ 18.2, 27.9, 29.0, 34.7, 43.5(C-4), 56.3(C-5), 62.2 (spiro), 76.3 (spiro), 108.5, 109.2, 110.3, 112.2, 115.7, 122.7, 125.3, 125.9, 128.2, 129.3, 132.9, 141.2, 141.4, 144.4, 152.5, 154.3, 176.8, 200.7 (C=O). Anal. Calcd. for C_26_H_24_N_2_O_4_: C 72.88, H 5.65, N 6.54; found: C 71.86, H 5.68, N 6.34.

### 3.2. Synthesis of 8-R-14-N-Methyl-2,14-diazaspiro[4.0.5.3]tetradecan-3,4-benzo-1,7-dione (General Method)

A solution of ketone **5**, **7** (5 mmol), isatin (0.88 g, 6 mmol), and sarcosine (0.45 g, 5 mmol) in isopropanol (20 mL) was heated at 60 °C for 3–5 h. The solvent was evaporated off at reduced pressure, and the solid residue was washed with hexane.

*14-N-Methyl-2,14-diazaspiro[4.0.5.3]tetradecan-3,4-benzo-1,7-dione* (**5**). Yield: 28%. m.p.: 71–74, ^1^H-NMR: δ 1.29–1.43 (m, 1H), 1.43–1.59 (m, 1H), 1.58–1.83 (m, 1H), 1.93 (ddd, 11.7, 7.9, 4.7, 1H), 1.98–2.02 (m, 1H), 2.05 (s. 1H), 2.07–2.15 (m, 1H), 2.22 (1H, ddd, *J* = 10.7, 8.5, 5.4), 2.36–2.46 (m, 1H), 2.66 (1H, ddd, *J* = 11.1, 8.3, 4.2), 3.19–3.30 (m, 2H), 6.86 (d, *J* = 7.7, 1H), 6.96 (d, 1H, *J* = 7.7), 7.01 (t, 1H, *J* = 7.7), 7.21 (t, *J* = 7.6, 1H), 8.57 (broad s, 1H). ^13^C-NMR: δ 21.5, 25.3, 34.0, 35.4, 35.6, 41.2, 51.9, 62.7 (spiro), 76.6 (spiro), 109.9, 122.6, 126.9, 127.1, 129.3, 141.5, 178.9, 212.0 (C=O). Anal. Calcd. for C_17_H_20_N_2_O_2_: C 71.81, H 7.09, N 9.85; found: C 71.43, H 7.12, N 9.71.

*8-Phenylmethyliden-14-N-methyl-2,14-diazaspiro[4.0.5.3]tetradecan-3,4-benzo-1,7-dione* (**7**). Yield: 34%. m.p.:123–126. ^1^H-NMR: δ 1.28–1.52 (m, 2H), 1.92 (1H, ddd, *J* = 14.2, 11.2, 3.1), 2.10 (s, 3H), 2.12–2.23 (m, 2H), 2.38–2.58 (m, 2H), 3.25 (ddd, *J* = 11.5, 5.6, 2.2, 1H), 3.49 (ddd, *J* = 9.3, 5.6, 3.6, 1H), 6.76 (*J* = 7.7, 1H), 6.91 (t, *J* = 7.5, 1H), 7.08–7.19 (m, 2H), 7.21–7.36 (m, 5H), 7.40 (s,1H), 8.00 (broad s, 1H). ^13^C-NMR: δ 21.6, 24.8, 34.5, 35.3, 35.7, 42.8, 50.8, 59.1 (spiro), 76.8 (spiro), 109.5, 115.8, 116.2, 122.6, 124.1, 126.9, 127.1, 129.3, 134.7, 141.5, 178.9, 202.3. Anal. Calcd. for C_24_H_24_N_2_O_2_: C 77.39, H 6.49, N 7.52; found: C 77.22, H 6.32, N 7.55.

## 4. Conclusions

For the first time, we have studied in the reactions of 1,3-dipolar cycloaddition of arylmethylidene cyclohexanones that are asymmetric due to peripheral substituents. It has been established that the interaction with sarcosine azomethine ylide proceeds with the participation of two non-equivalent centers of the dipolarophile. On the basis of a set of NMR spectroscopy data, it is established that the reaction products indicate the *endo*-selectivity of the dipole addition. Also, for the first time, β-amino ketones (Mannich bases) were used as dipolarophile equivalents of unsaturated ketones. It was found that the interaction proceeds diastereoselectively through the generated center, with the formation of *endo*-adducts.

## Figures and Tables

**Table 1:** Yields and isomer ratios of compounds **2** and **3**.

Compound	Ratio 2/3	Yields, %	Ratio *Endo*/*Exo*
a	64/36	78	100/0
b	50/50	72	100/0
c	55/45	82	100/0
d	80/20	65	100/0
e	80/20	68	100/0
f	70/30	64	100/0

## Supplementary Materials

Spectral data for all new products and NOESY spectra for compound **2**, **3a**–**f** and **5** are available online.
