# Supplementary material for: A Diastereoselective Synthesis of Dispiro[oxindole-cyclohexanone]pyrrolidines by 1,3-Dipolar Cycloaddition
Source: Molecules. 2017 Dec 4;22(12):2134. doi: 10.3390/molecules22122134 (PMC6149807; doi:10.3390/molecules22122134)
Supplement: Supplementary file 1 [file molecules-22-02134-s001.pdf]

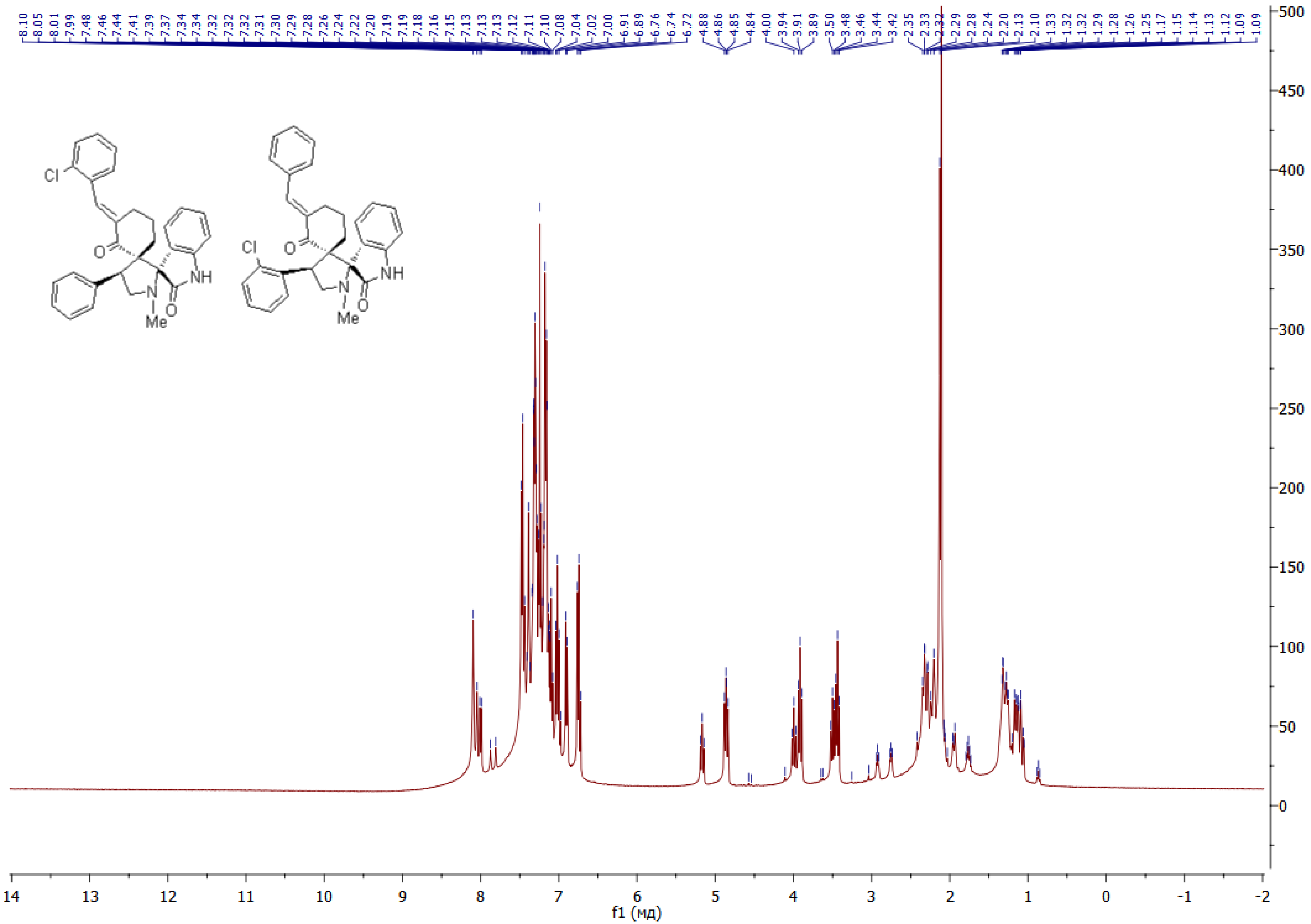


The $^{1}$H NMR 1-N-methyl-spiro[2.3$^{1}$]oxindole-spiro[3.2$^{11}$]611-(2-chlorophenyl)methylidenecyclohexanone-4-phenyl-pyrrolidines and 1-N-methyl-spiro[2.3$^{1}$]oxindole-spiro[3.2$^{11}$]611-phenylmethylidenecyclohexanone-4- (2-chlorophenyl) pyrrolidines (**2 and 3 a**)

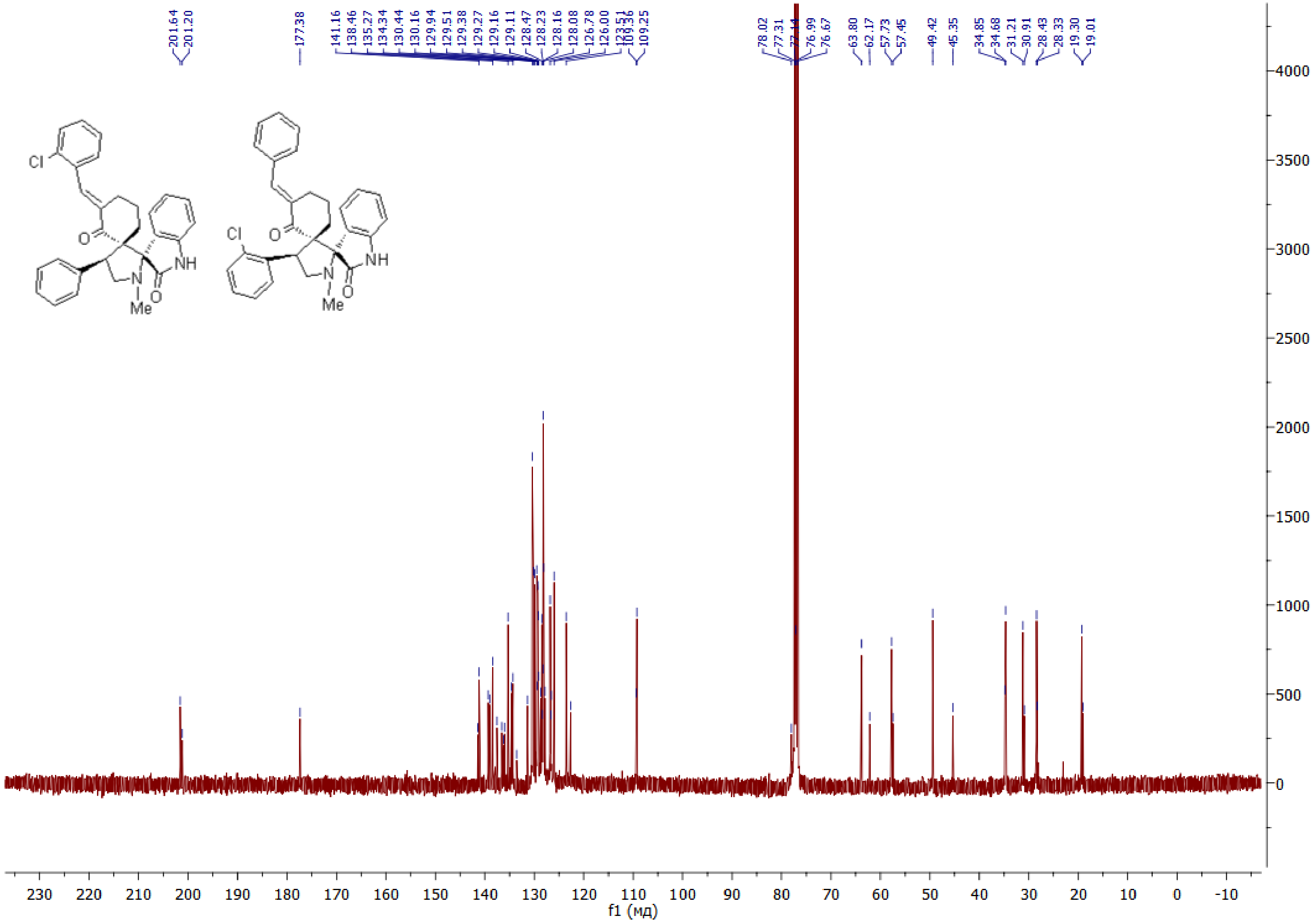


The $^{13}$C NMR 1-N-methyl-spiro[2.3$^{1}$]oxindole-spiro[3.2$^{11}$]611-(2-chlorophenyl)methylidenecyclohexanone-4-phenyl-pyrrolidines and 1-N-methyl-spiro[2.3$^{1}$]oxindole-spiro[3.2$^{11}$]611-phenylmethylidenecyclohexanone-4- (2-chlorophenyl) pyrrolidines (**2** and **3 a**)

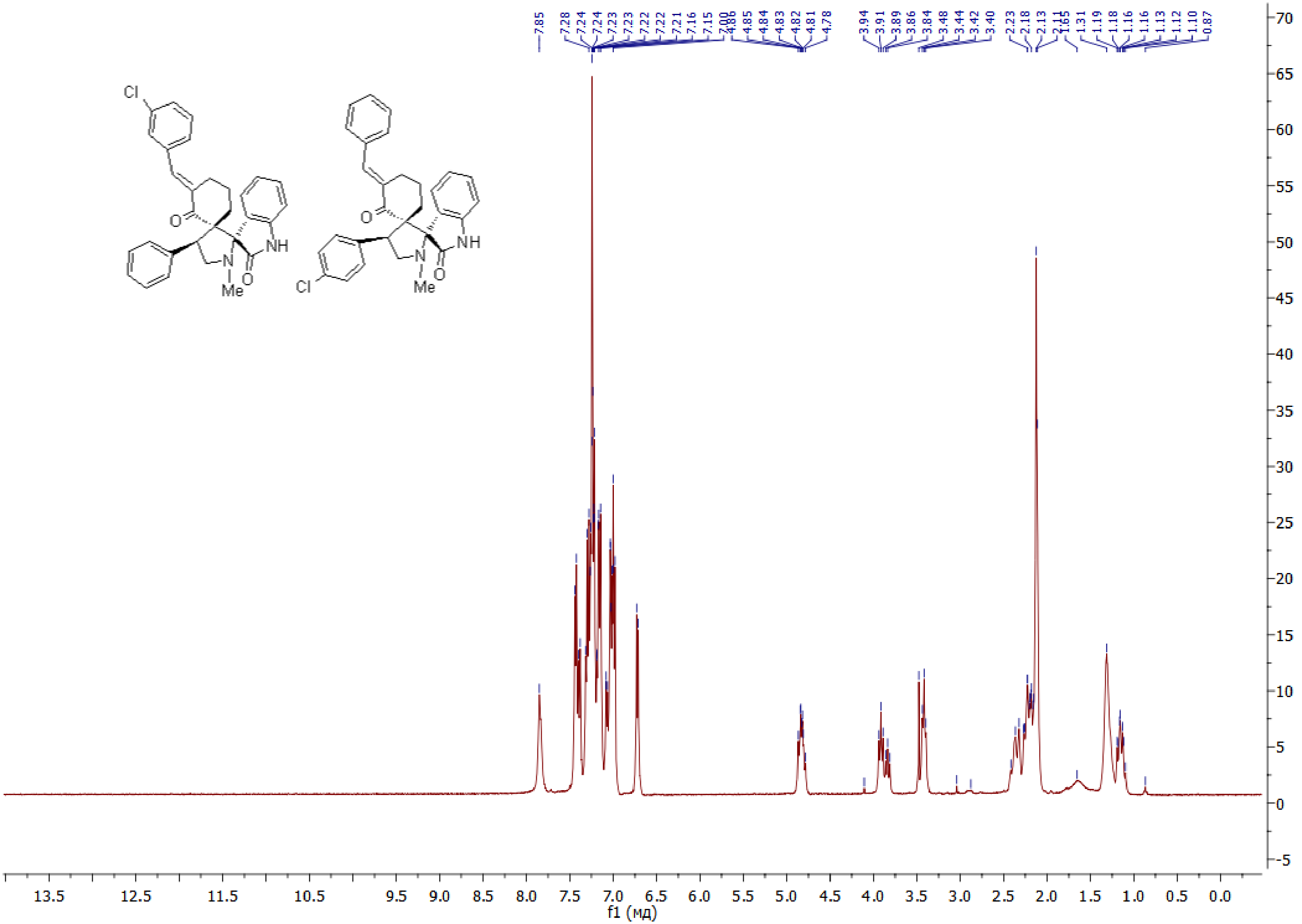


The $^{1}$H NMR 1-N-methyl-spiro[2.3$^{1}$]oxindole-spiro[3.2$^{11}$]611-(4-chlorophenyl)methylidenecyclohexanone-4-phenyl-pyrrolidines and 1-N-methyl-spiro[2.3$^{1}$]oxindole-spiro[3.2$^{11}$]611-phenylmethylidenecyclohexanone-4- (4-chlorophenyl)pyrrolidines (**2** and **3 b**)

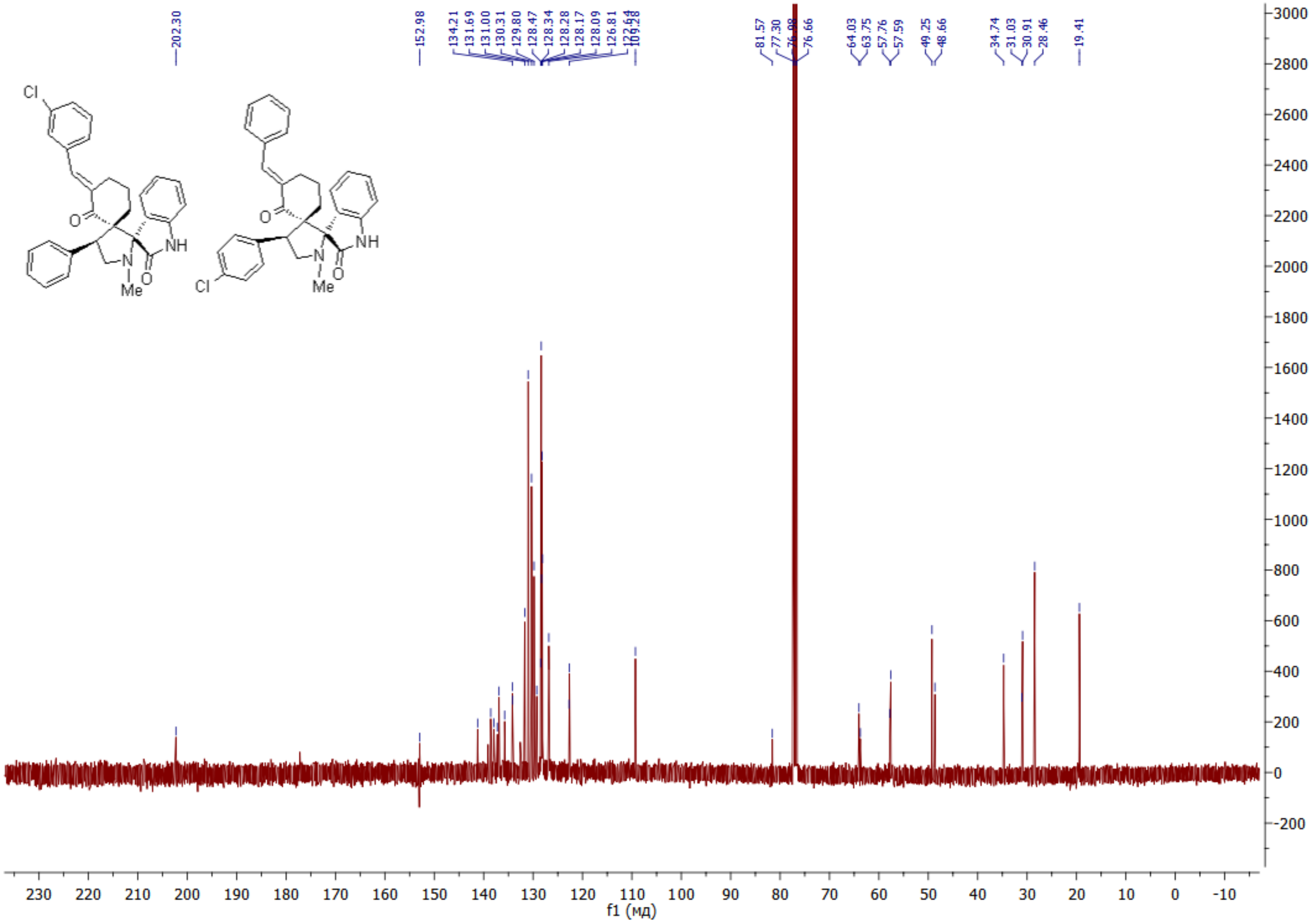

The $^{13}$C NMR 1-N-methyl-spiro[2.3[1]]oxindole-spiro[3.2[11]]611-(4-chlorophenyl)methylidenecyclohexanone-4-phenyl-pyrrolidines and 1-N-methyl-spiro[2.3[1]]oxindole-spiro[3.2[11]]611-phenylmethylidenecyclohexanone-4- (4-chlorophenyl)pyrrolidines (**2** and **3 b**)

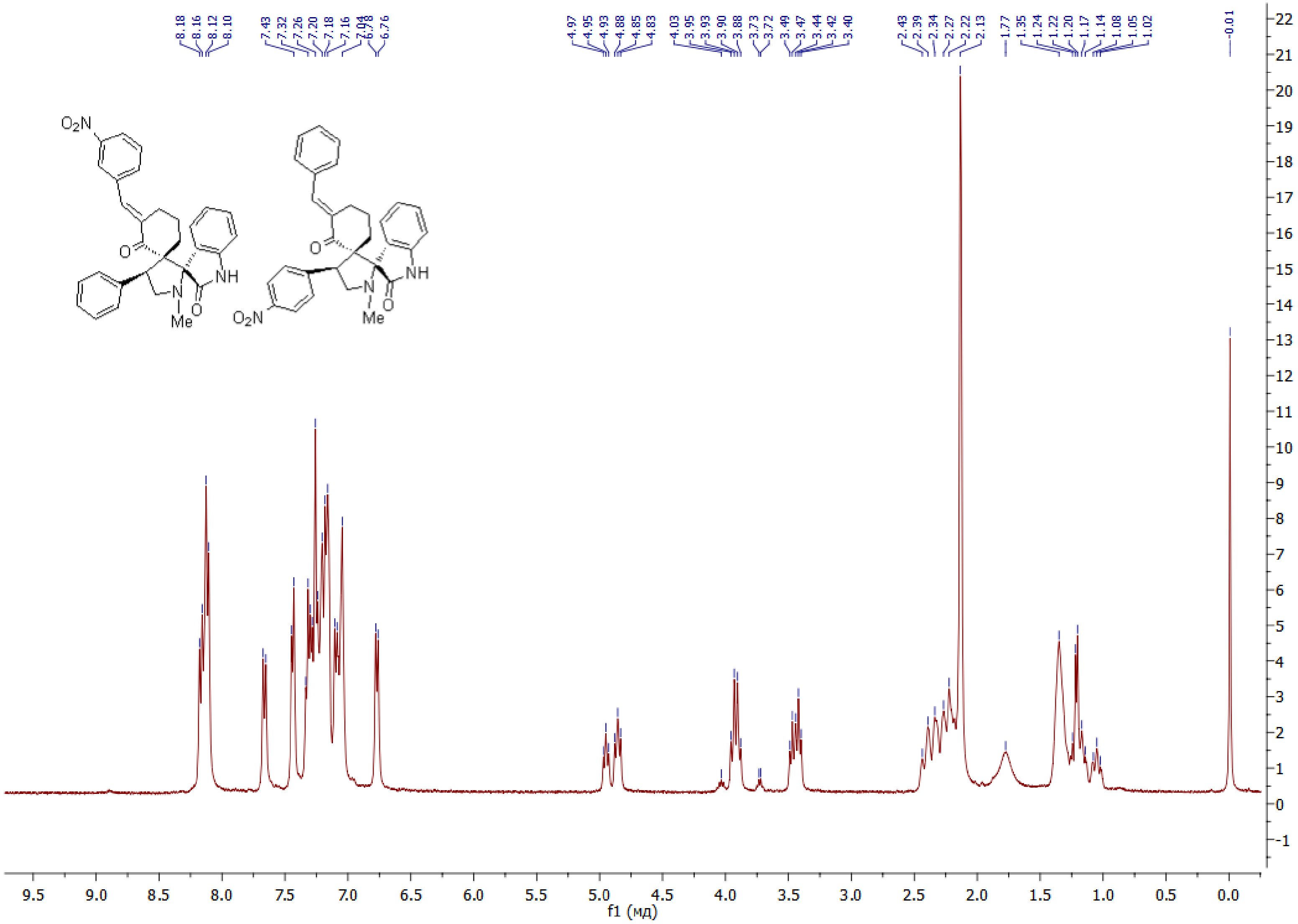

The $^{1}$H NMR 1-N-methyl-spiro[2.3$^{1}$]oxindole-spiro[3.2$^{11}$]6$^{11}$-(4-nitrophenyl)methylidenecyclohexanone-4-phenyl-pyrrolidines and 1-N-methyl-spiro[2.3$^{1}$]oxindole-spiro[3.2$^{11}$]6$^{11}$-phenylmethylidenecyclohexanone-4- (4-nitrophenyl) -pyrrolidines (**2** and **3 c**)

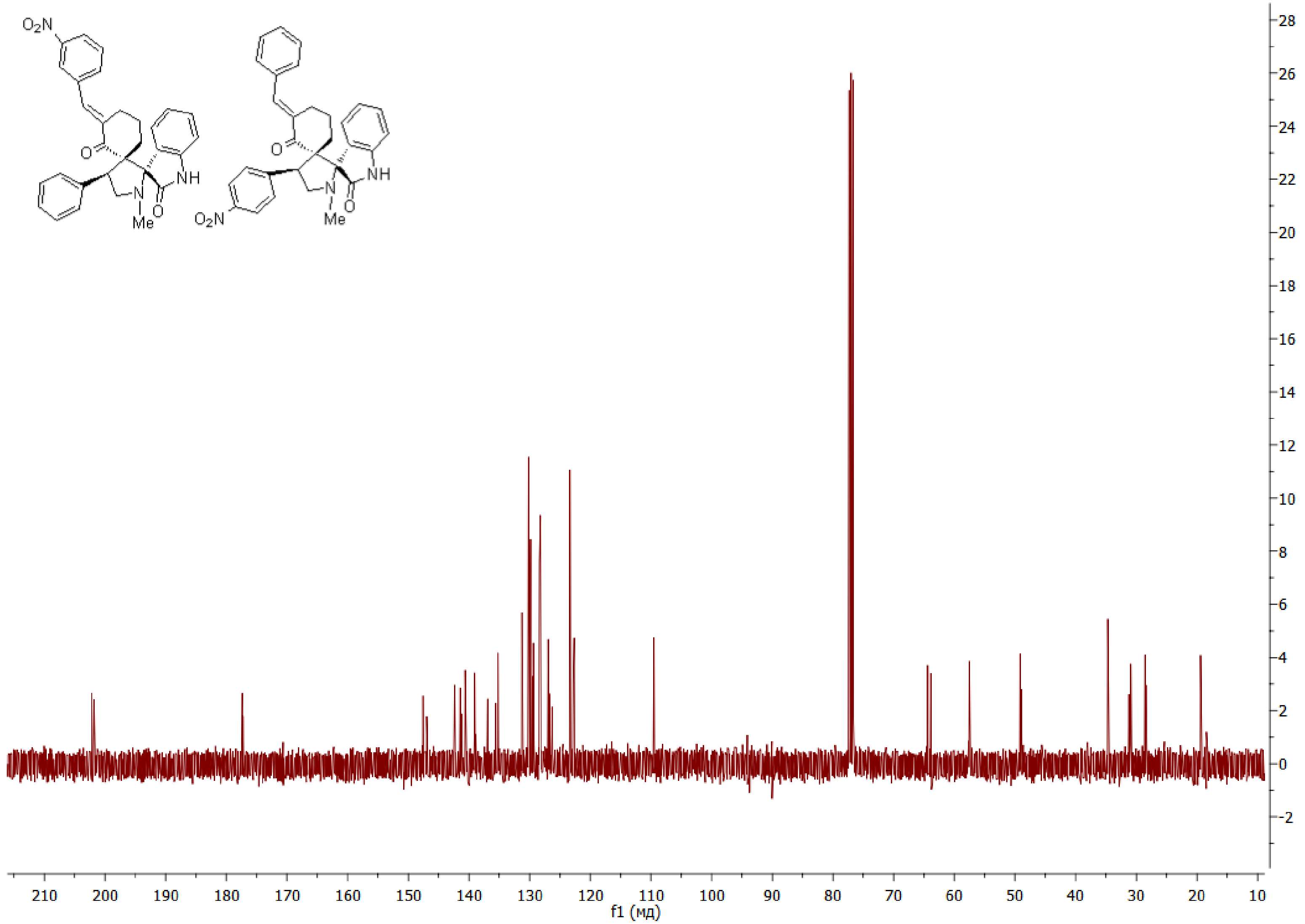


The $^{13}$C NMR 1-N-methyl-spiro[2.$3^1$]oxindole-spiro[3.$2^{11}$]$6^{11}$-(4-nitrophenyl)methylidenecyclohexanone-4-phenyl-pyrrolidines and 1-N-methyl-spiro[2.$3^1$]oxindole-spiro[3.$2^{11}$]$6^{11}$-phenylmethylidenecyclohexanone-4- (4-nitrophenyl) -pyrrolidines (**2** and **3 c**)

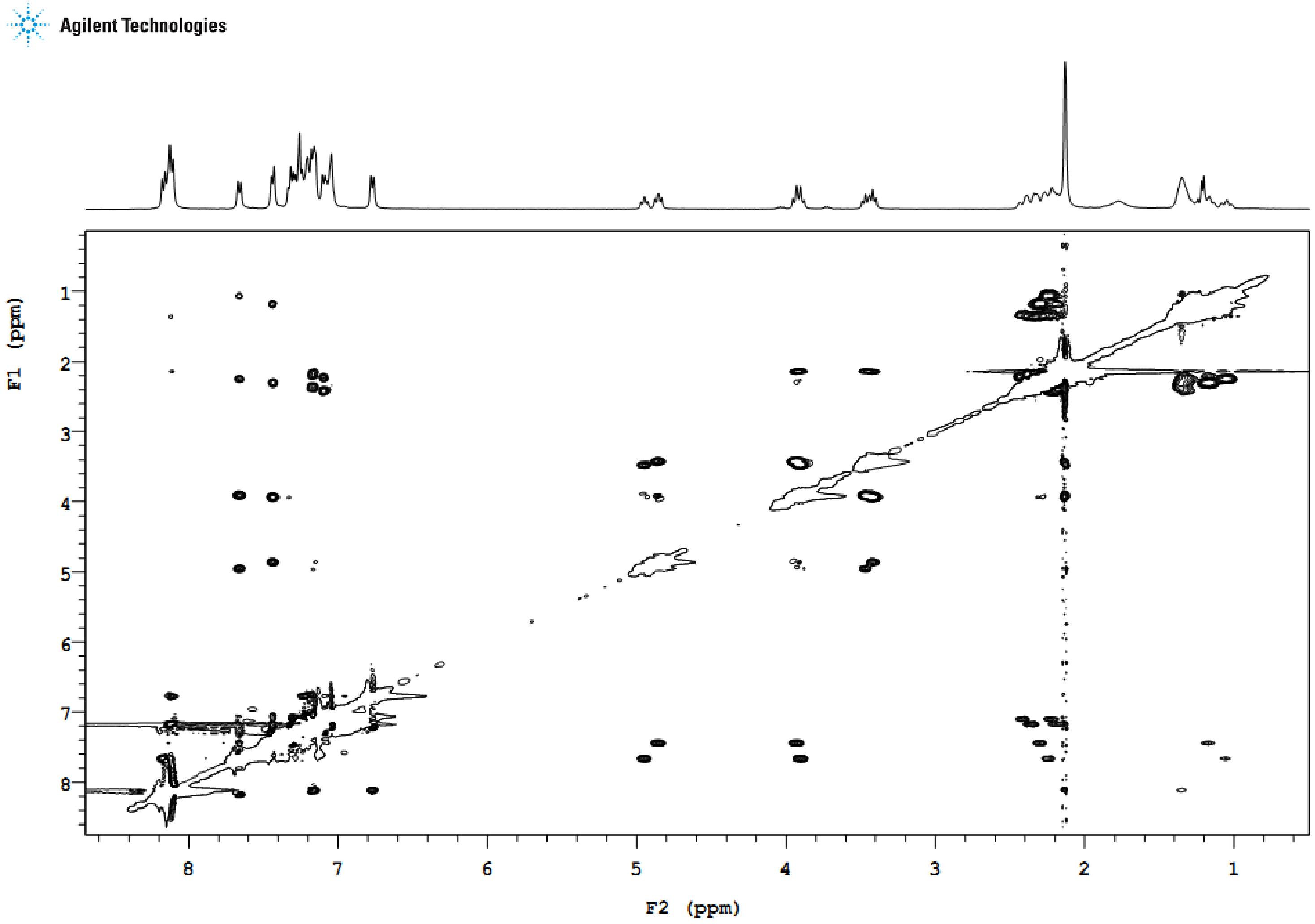


The NOESY spectrum of 1-N-methyl-spiro[2.3[1]]oxindole-spiro[3.2[11]]6[11]-(4-nitrophenyl)methylidenecyclohexanone-4-phenyl-pyrrolidines and 1-N-methyl-spiro[2.3[1]]oxindole-spiro[3.2[11]]6[11]-phenylmethylidenecyclohexanone-4- (4-nitrophenyl) -pyrrolidines (**2** and **3 c**)

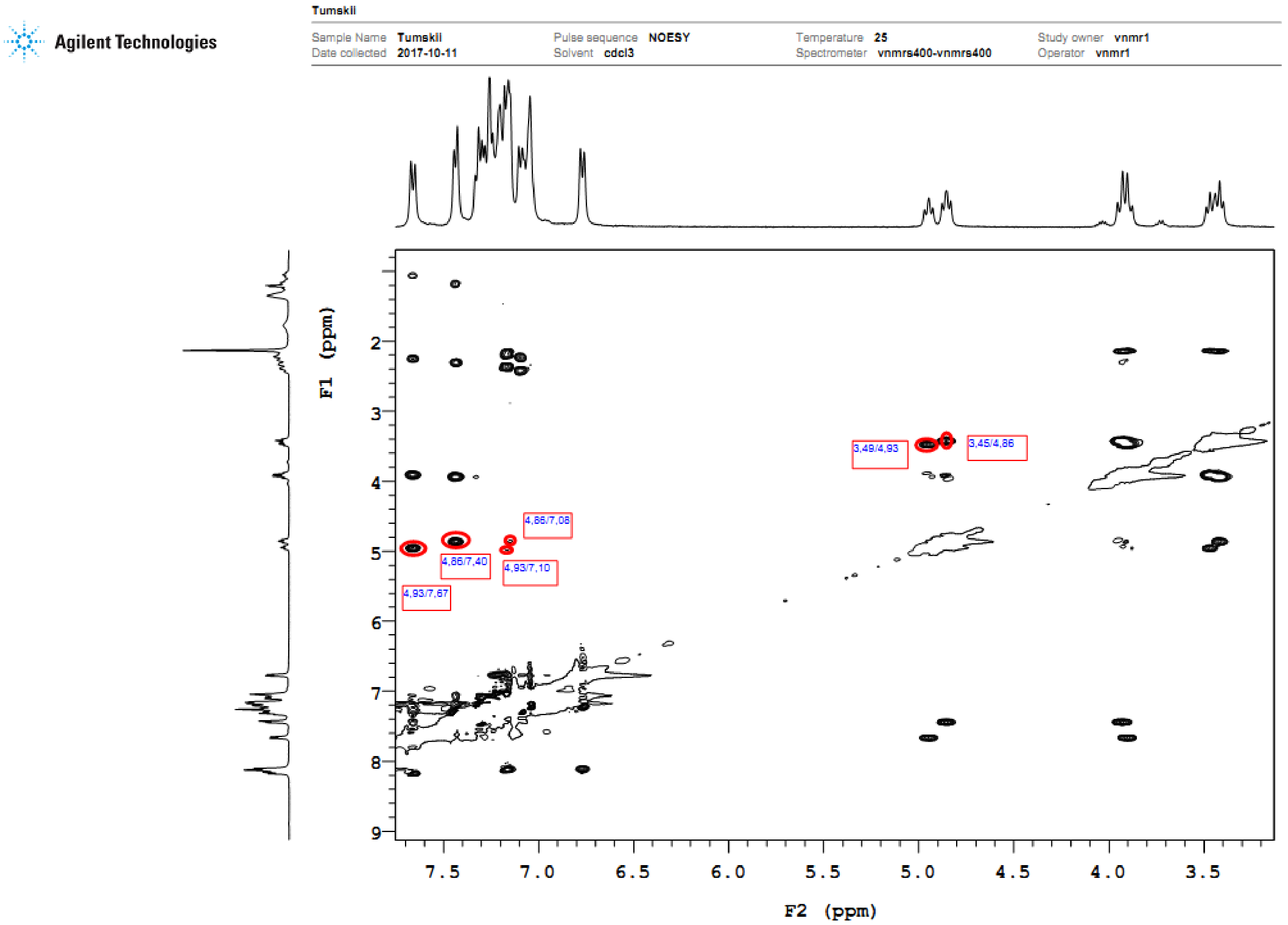


The detailed NOESY spectrum of 1-N-methyl-spiro$[2.3^{1}]$oxindole-spiro$[3.2^{11}]6^{11}$-(4-nitrophenyl)methylidenecyclohexanone-4-phenyl-pyrrolidines and 1-N-methyl-spiro$[2.3^{1}]$oxindole-spiro$[3.2^{11}]6^{11}$-phenylmethylidenecyclohexanone-4- (4-nitrophenyl) -pyrrolidines (**2** and **3 c**)

Agilent Technologies

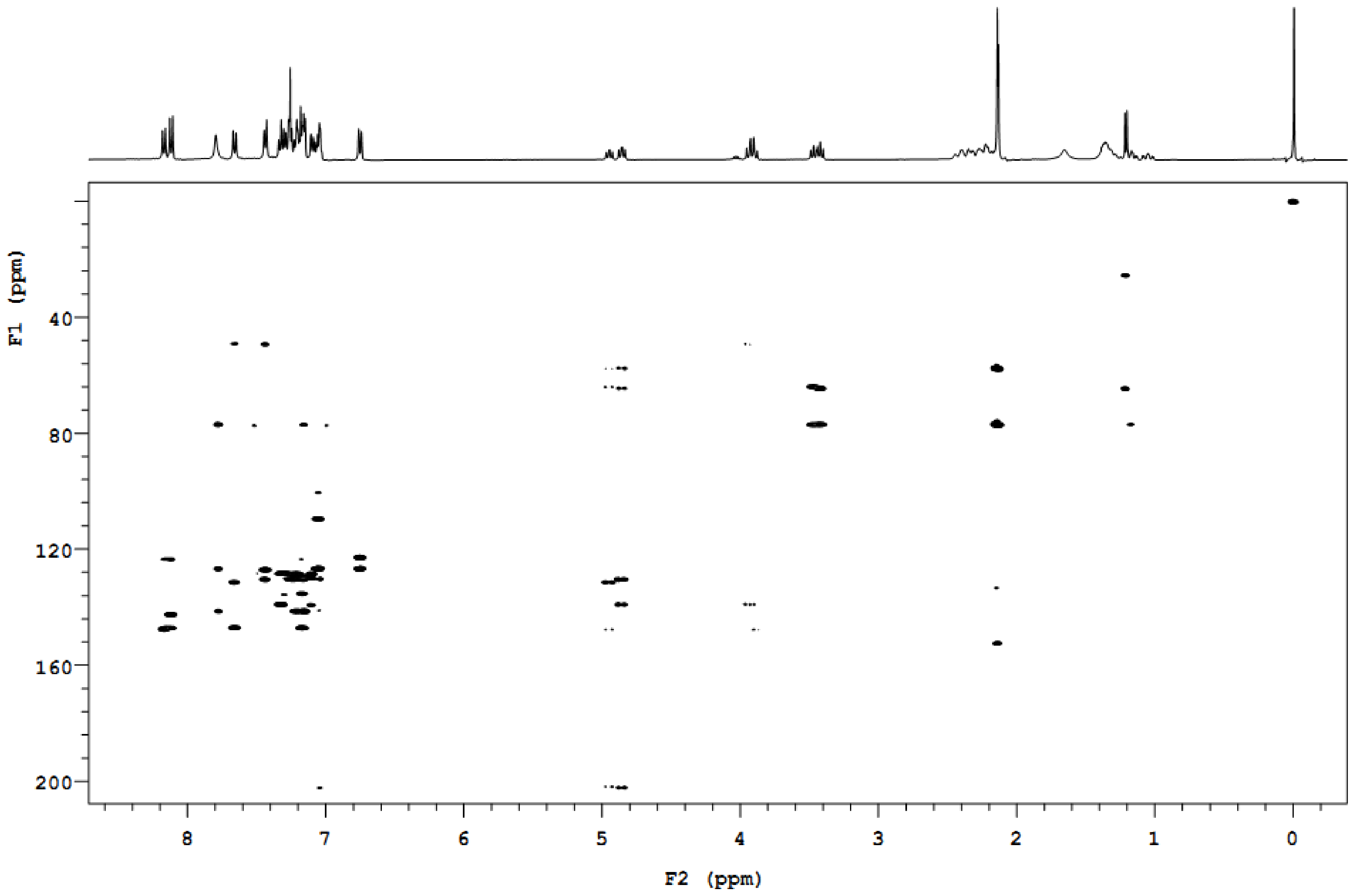


The $^{1}H^{13}C$ HMBC spectrum of 1-N-methyl-spiro[2.3$^{1}$]oxindole-spiro[3.2$^{11}$]6$^{11}$-(4-nitrophenyl)methylidenecyclohexanone-4-phenyl-pyrrolidines and 1-N-methyl-spiro[2.3$^{1}$]oxindole-spiro[3.2$^{11}$]6$^{11}$-phenylmethylidenecyclohexanone-4- (4-nitrophenyl) -pyrrolidines (**2** and **3 c**)

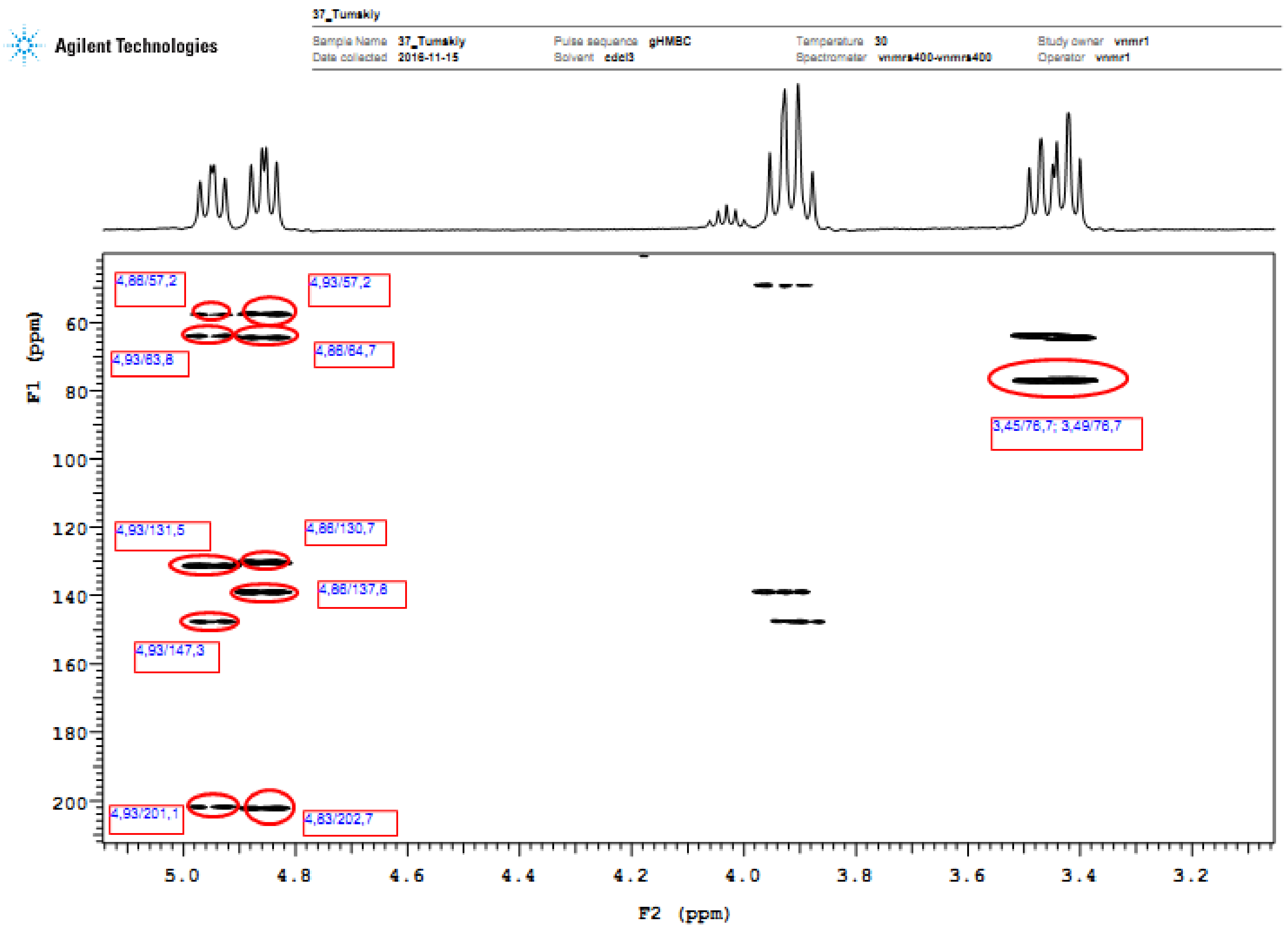


The detailed $^{1}H^{13}C$ HMBC spectrum of 1-N-methyl-spiro[2.3$^{1}$]oxindole-spiro[3.2$^{11}$]6$^{11}$-(4-nitrophenyl)methylidenecyclohexanone-4-phenyl-pyrrolidines and 1-N-methyl-spiro[2.3$^{1}$]oxindole-spiro[3.2$^{11}$]6$^{11}$-phenylmethylidenecyclohexanone-4- (4-nitrophenyl) -pyrrolidines (**2** and **3 c**)

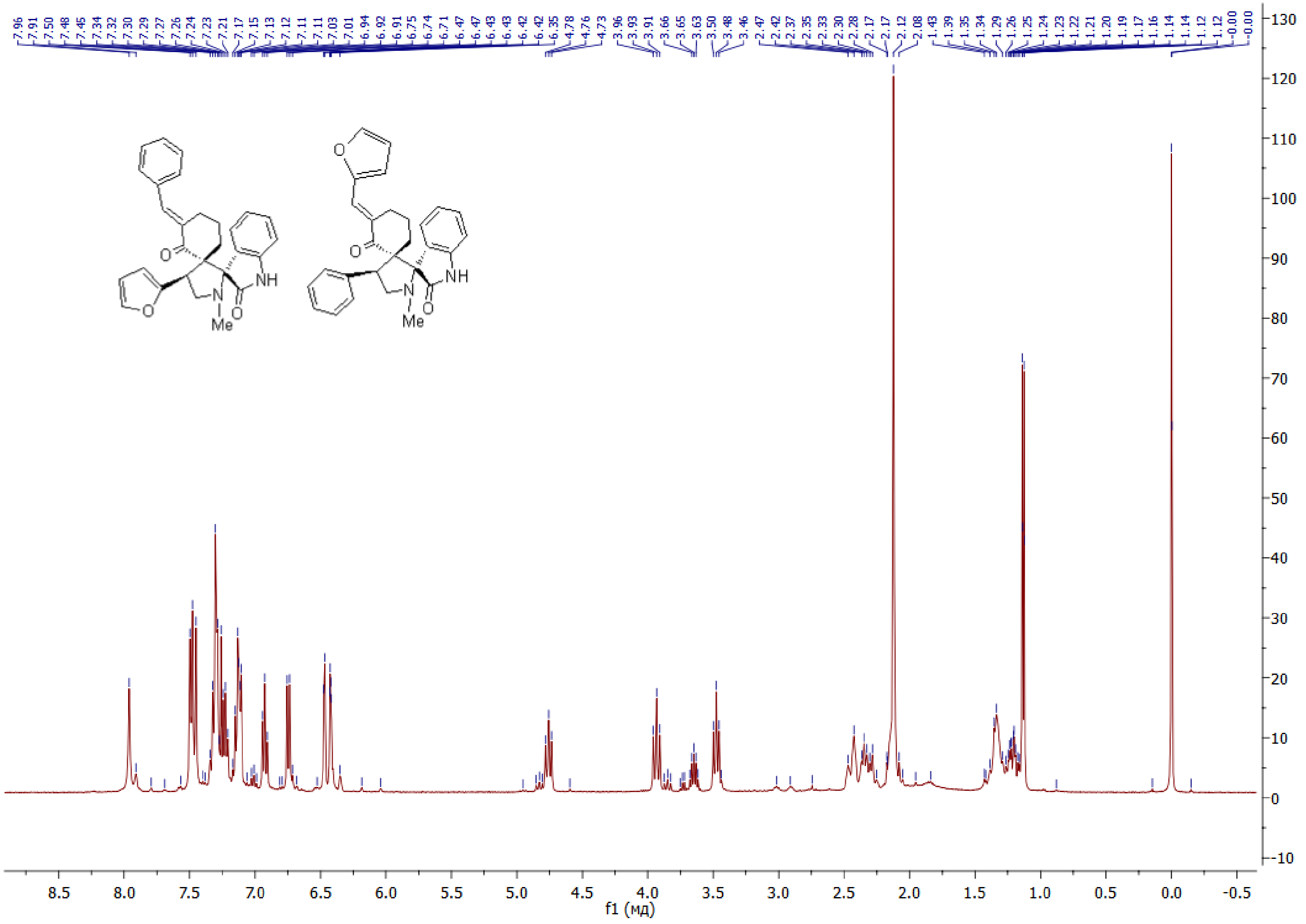


The $^{1}$H NMR 1-N-methyl-spiro[2.3$^{1}$]oxindole-spiro[3.2$^{11}$]6$^{11}$-2-furylmethylidenecyclohexanone-4-phenyl-pyrrolidines and 1-N-methyl-spiro[2.3$^{1}$]oxindole-spiro[3.2$^{11}$]6$^{11}$-phenylmethylidenecyclohexanone-4-2-furylpyrrolidines (**3** and **4 d**)

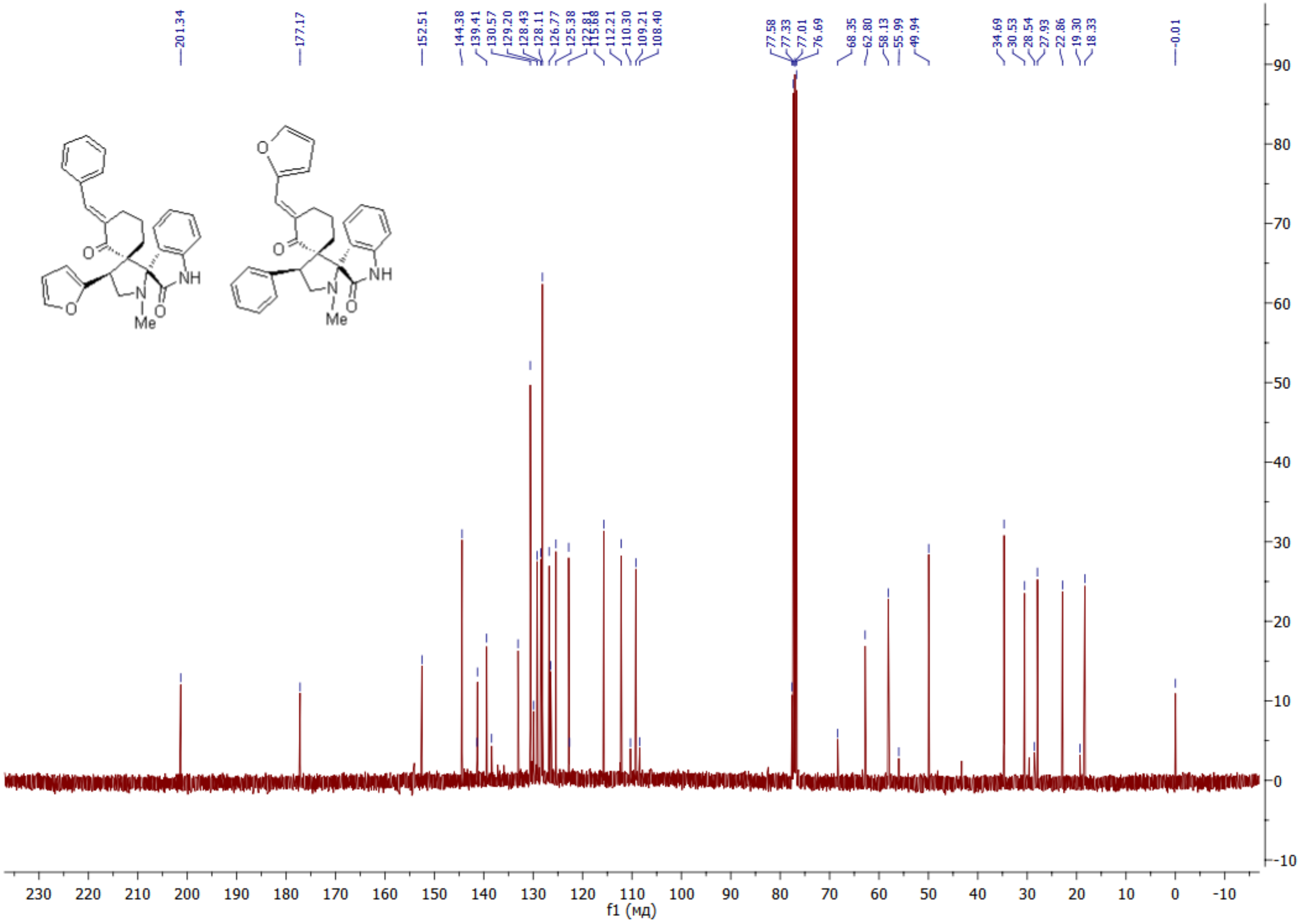


The $^{13}$C NMR 1-N-methyl-spiro[2.3$^{1}$]oxindole-spiro[3.2$^{11}$]6$^{11}$-2-furylmethylidenecyclohexanone-4-phenyl-pyrrolidines and 1-N-methyl-spiro[2.3$^{1}$]oxindole-spiro[3.2$^{11}$]6$^{11}$-phenylmethylidenecyclohexanone-4-2-furylpyrrolidines (**3** and **4 d**)

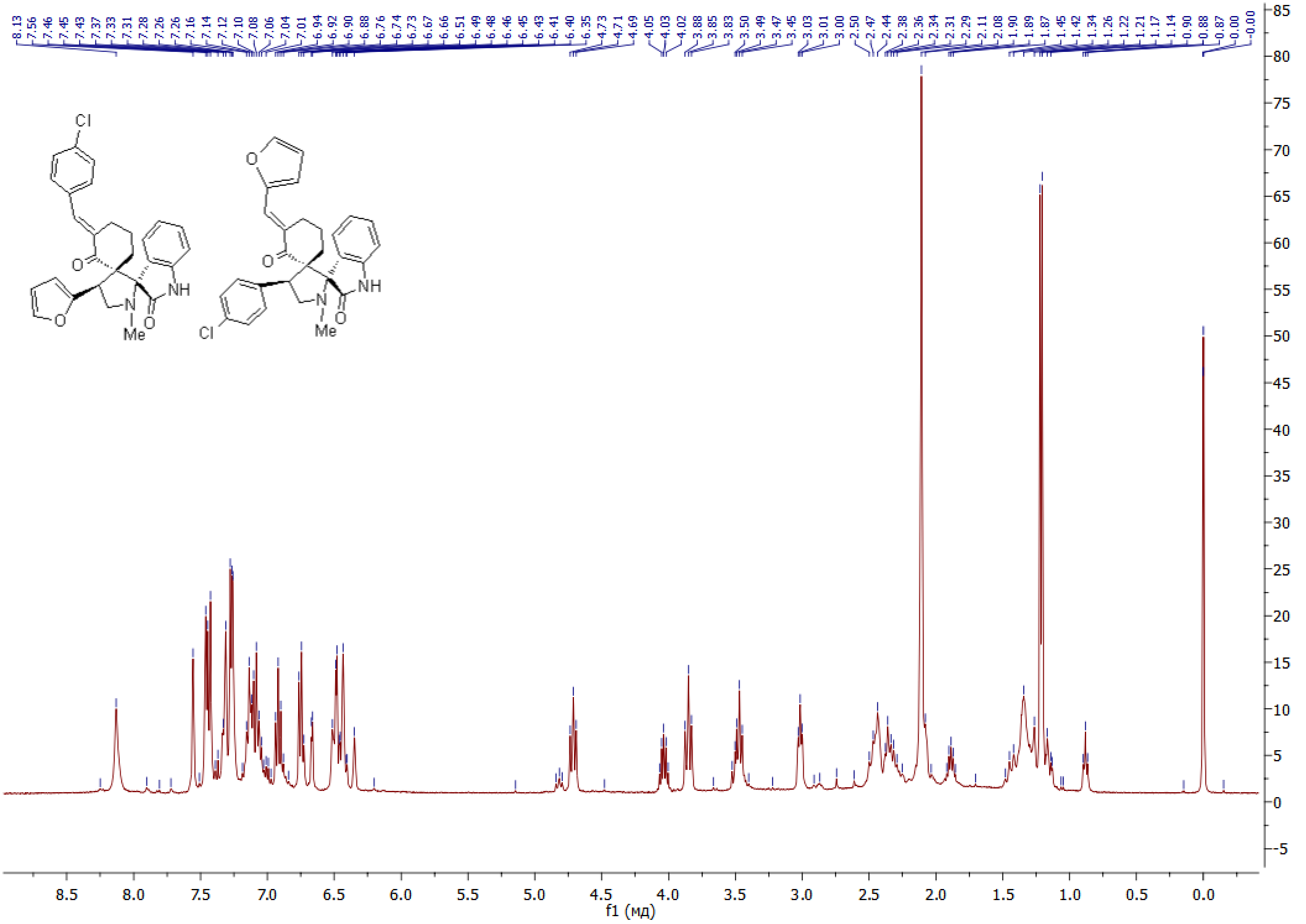


The $^{1}$H NMR 1-N-methyl-spiro[2.3$^{1}$]oxindole-spiro[3.2$^{11}$]6$^{11}$-2-furylmethylidenecyclohexanone-4-4-chlorophenyl-pyrrolidines and 1-N-methyl-spiro[2.3$^{1}$]oxindole-spiro[3.2$^{11}$]6$^{11}$--4-chlorophenylmethylidenecyclohexanone- 4-2-furylpyrrolidines (**3** and **4 e**)

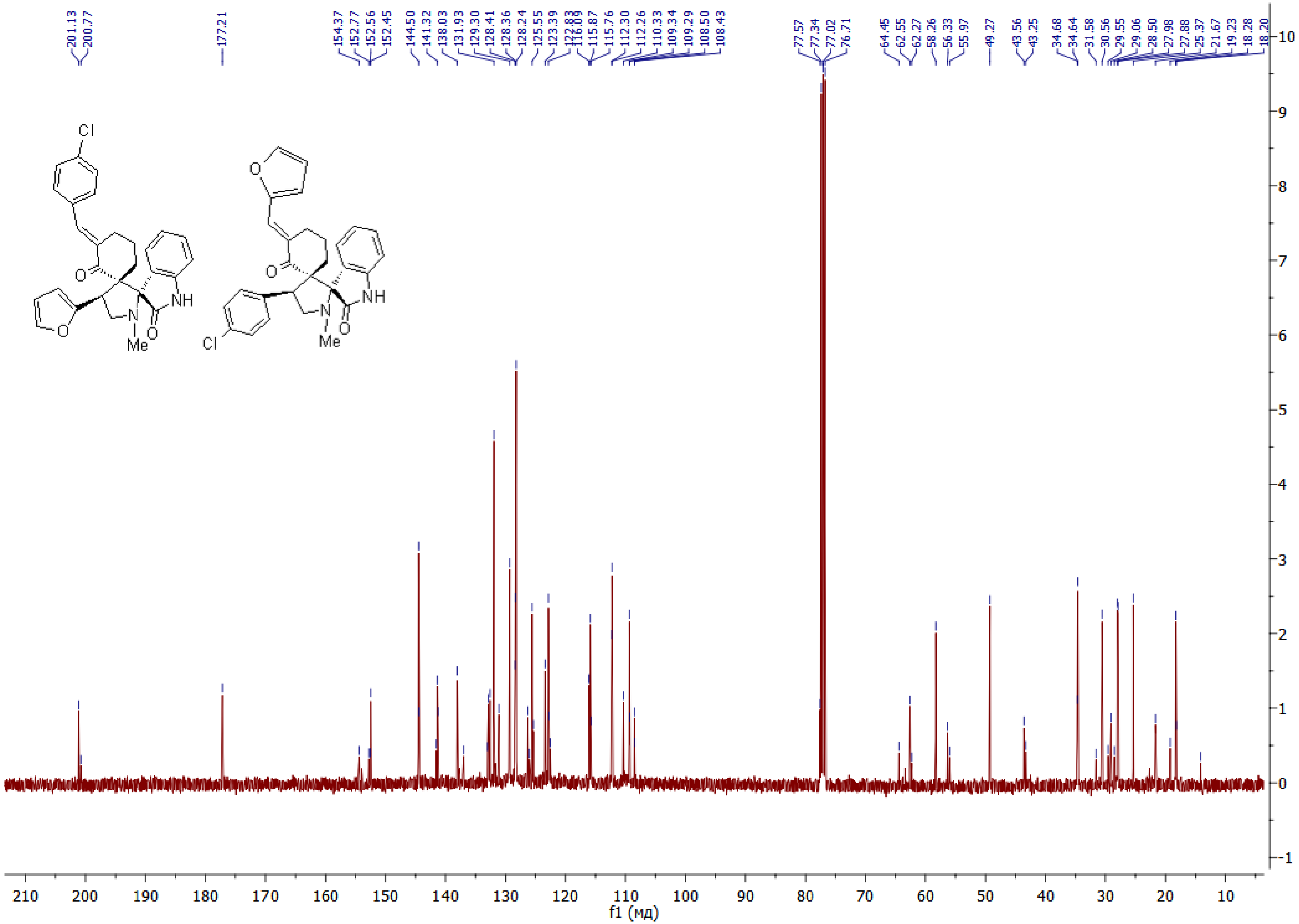


The $^{13}$C NMR 1-N-methyl-spiro[2.3$^{1}$]oxindole-spiro[3.2$^{11}$]6$^{11}$-2-furylmethylidenecyclohexanone-4-4-chlorophenyl-pyrrolidines and 1-N-methyl-spiro[2.3$^{1}$]oxindole-spiro[3.2$^{11}$]6$^{11}$--4-chlorophenylmethylidenecyclohexanone- 4-2-furylpyrrolidines (**3** and **4 e**)

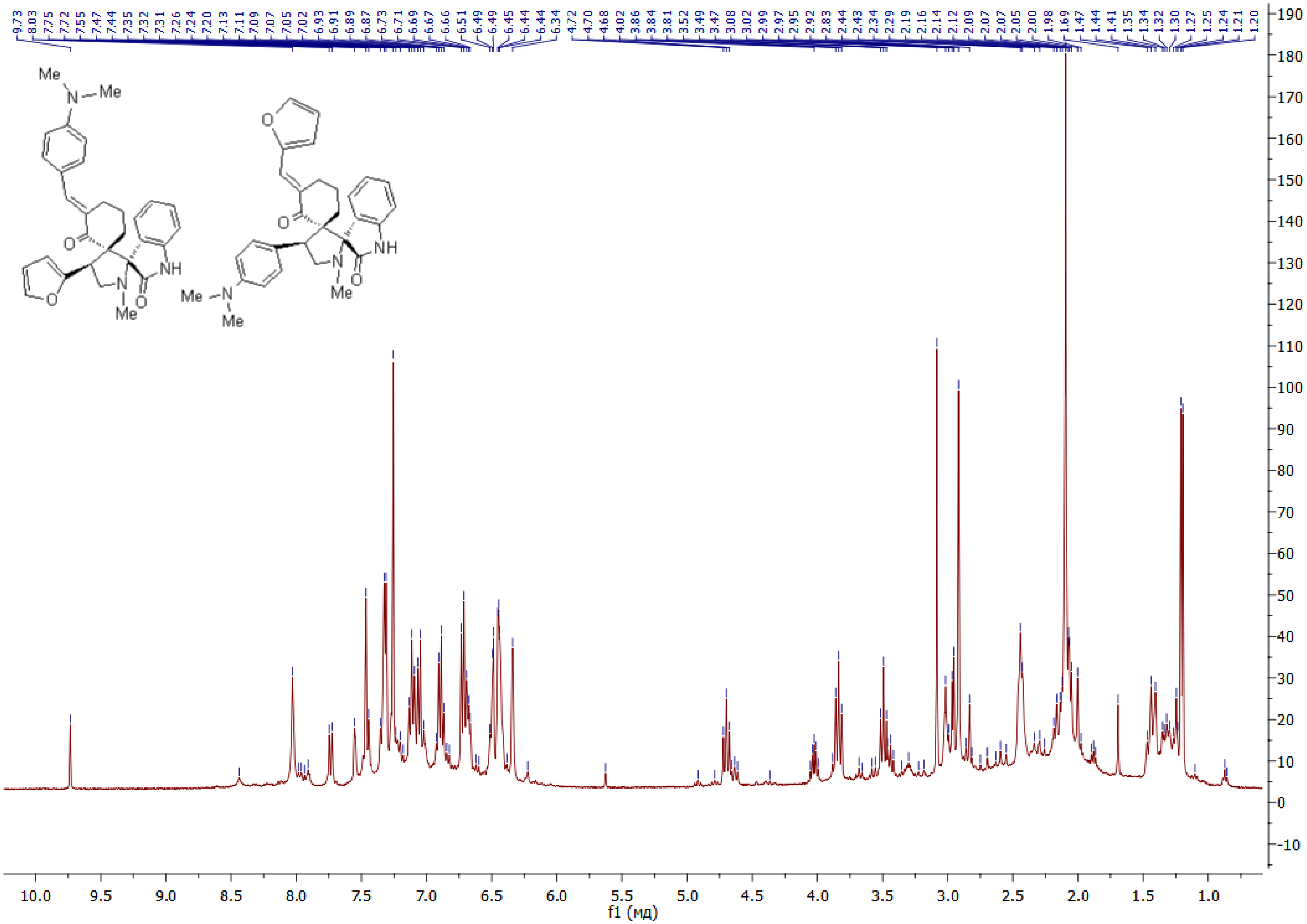

The $^{1}$H NMR  1-N-methyl-spiro[2.31]oxindole-spiro[3.211]611-2-furylmethylidenecyclohexanone-4-4-N,N-(dimethyl)phenyl-pyrrolidines and 1-N-methyl-spiro[2.31]oxindole-spiro[3.211]611--4-N,N-(dimethyl)phenylmethylidenecyclohexanone- 4-2-furylpyrrolidines (**2** and **3 f**)

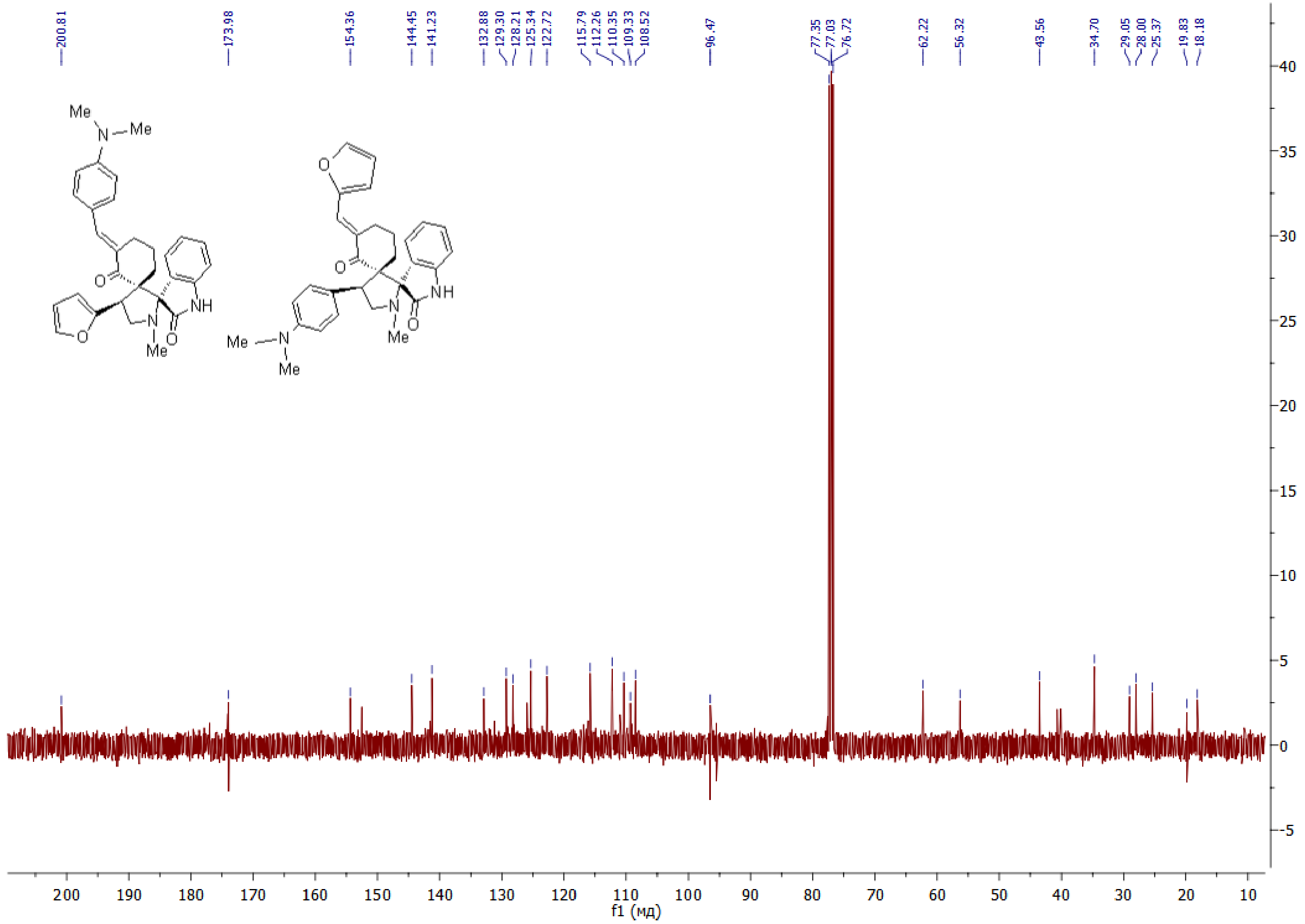

The $^{13}C$ NMR  1-N-methyl-spiro[2.31]oxindole-spiro[3.211]611-2-furylmethylidenecyclohexanone-4-4-N,N-(dimethyl)phenyl-pyrrolidines and 1-N-methyl-spiro[2.31]oxindole-spiro[3.211]611--4-N,N-(dimethyl)phenylmethylidenecyclohexanone- 4-2-furylpyrrolidines (2 and 3 f)

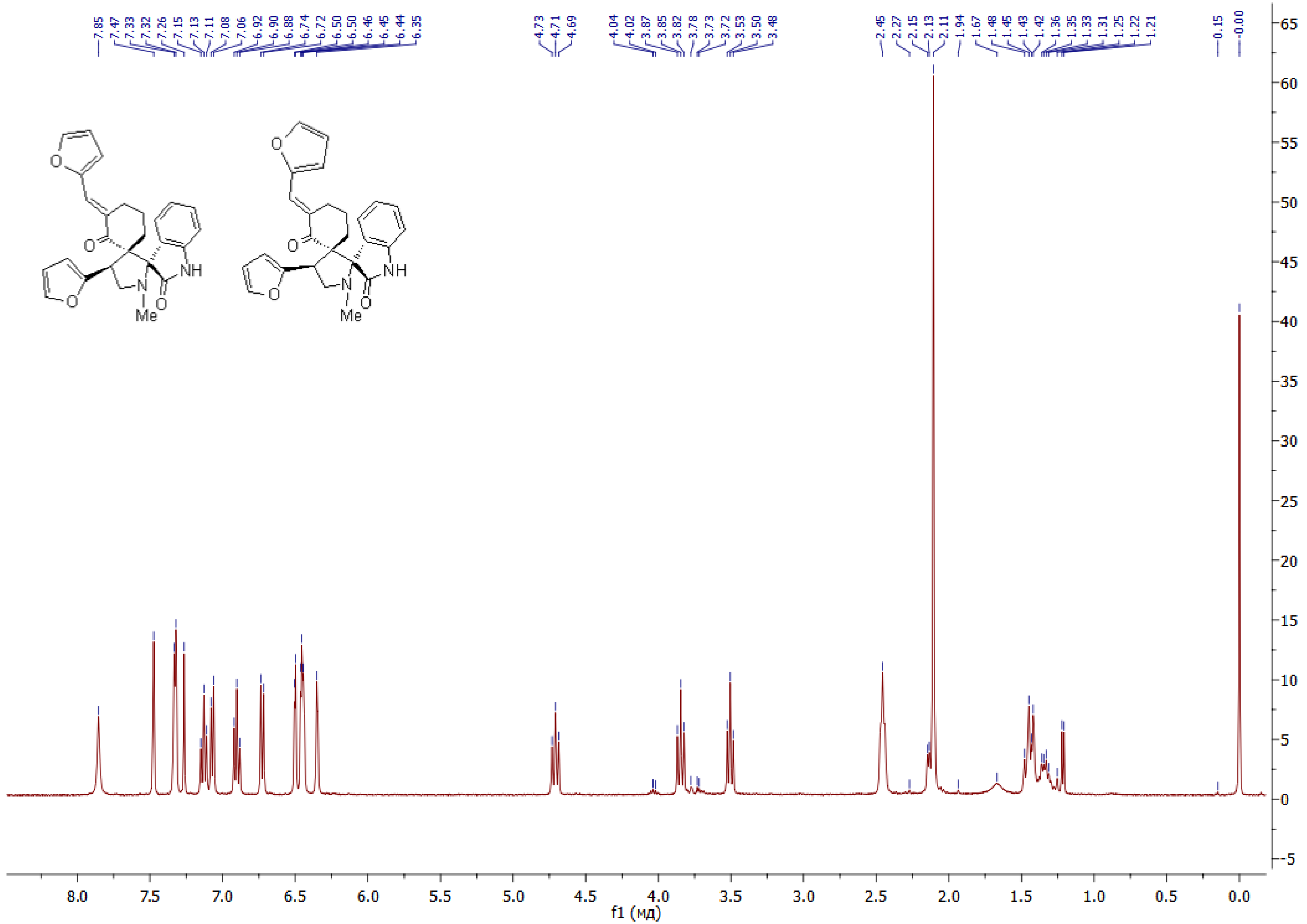


The $^{1}$H NMR 1-N-methyl-spiro[2.3$^{1}$]oxindole-spiro[3.2$^{11}$]6$^{11}$-2-furylmethylidenecyclohexanone-4-2-furylpyrrolidines (**3g)**

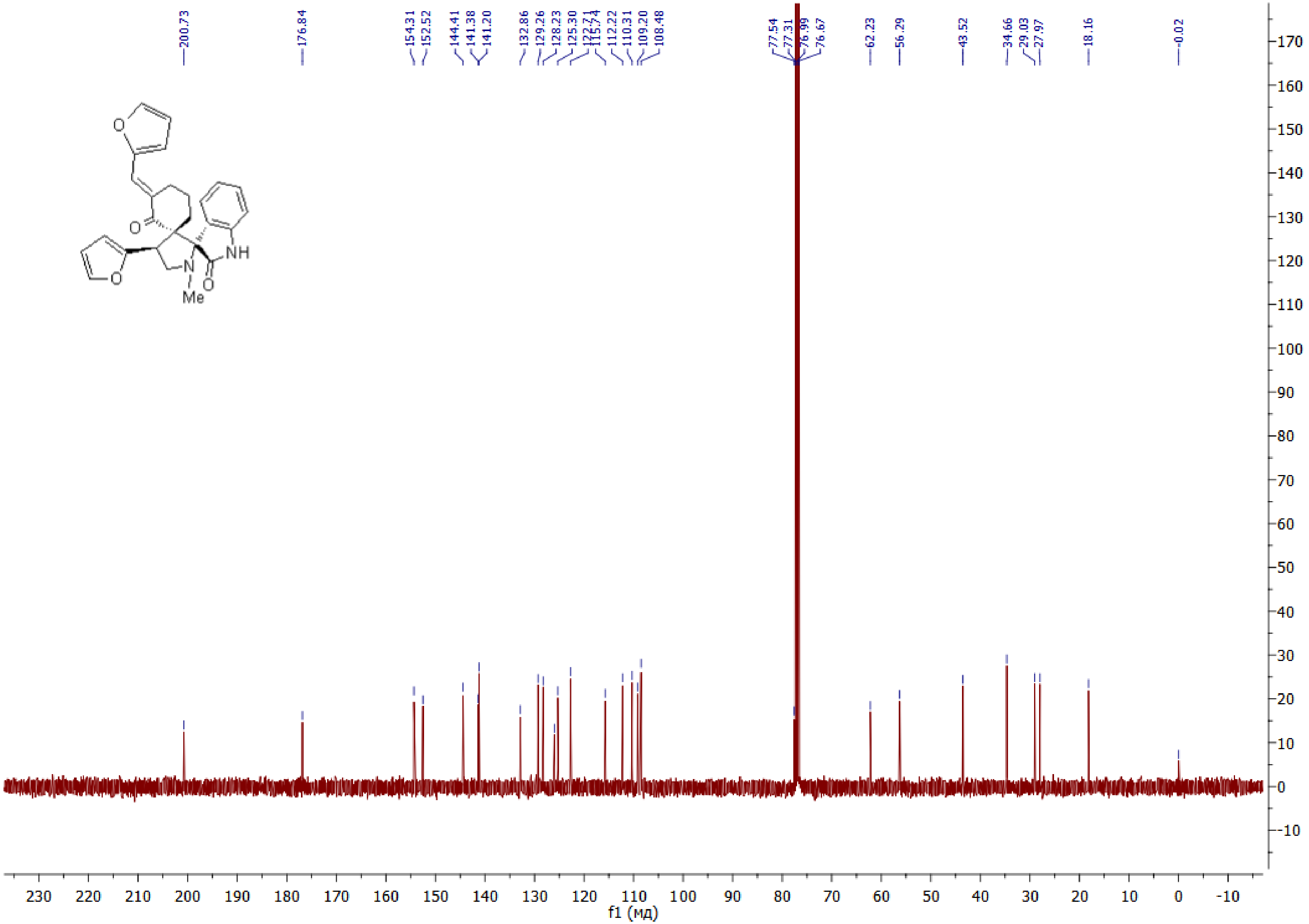


The $^{13}$C NMR 1-N-methyl-spiro[2.$3^{1}$]oxindole-spiro[3.$2^{11}$]$6^{11}$-2-furylmethylidenecyclohexanone-4-2-furylpyrrolidines (**3f)**

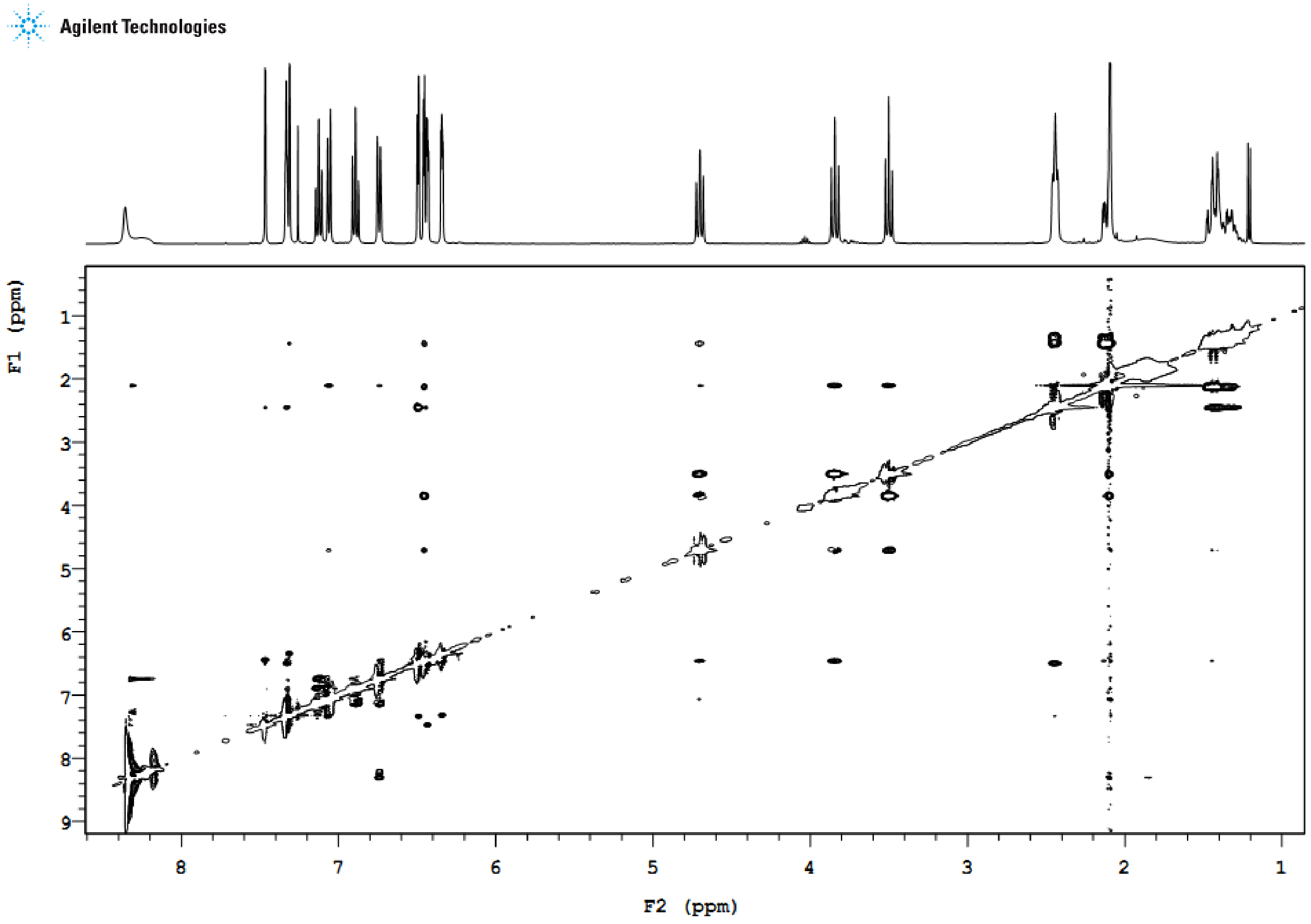


The NOESY spectrum 1-N-methyl-spiro[2.3[1]]oxindole-spiro[3.2[11]]6[11]-2-furylmethylidenecyclohexanone-4-2-furylpyrrolidines (**3f)**

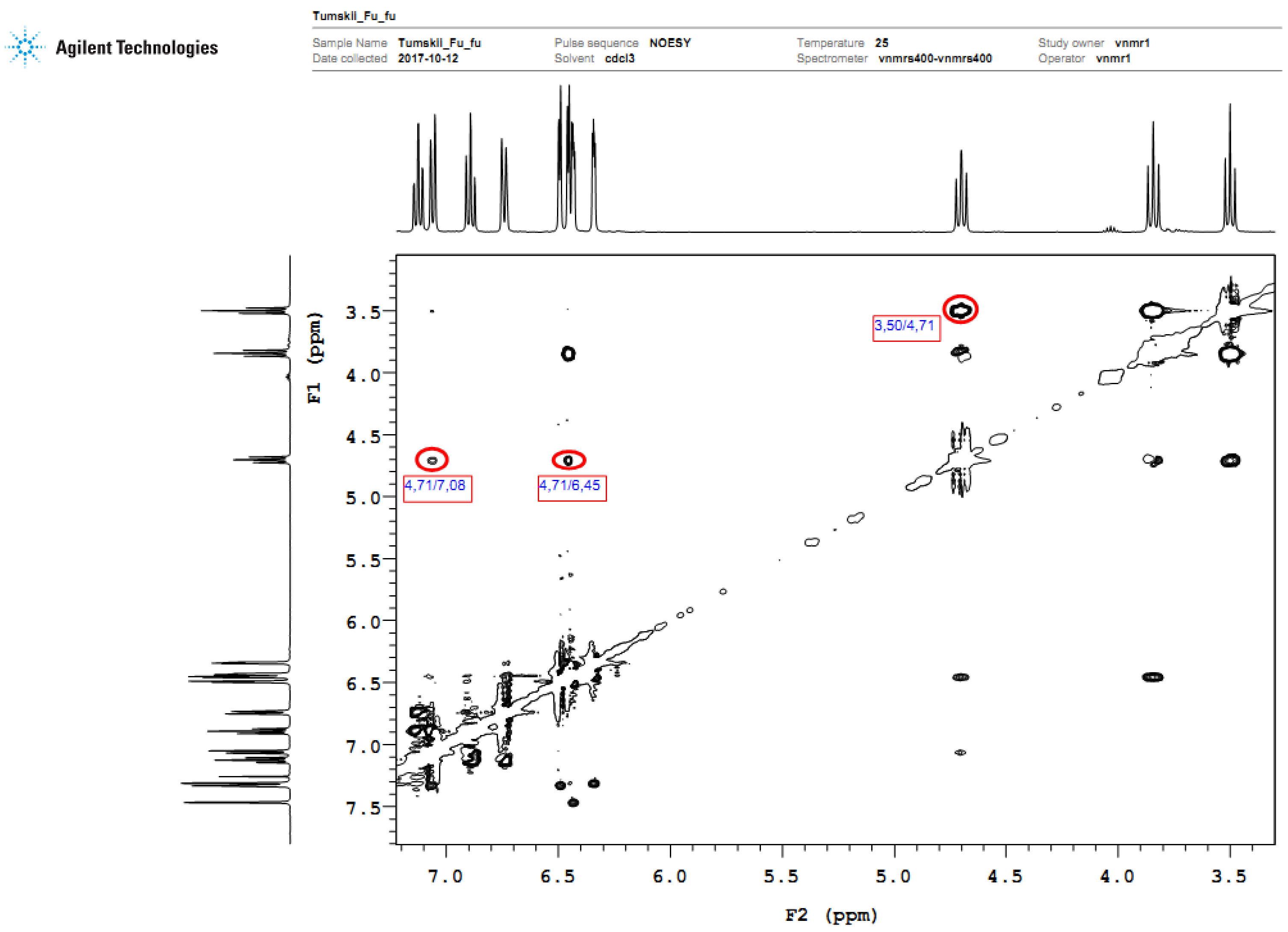


The detailed NOESY spectrum 1-N-methyl-spiro[2.3$^{1}$]oxindole-spiro[3.2$^{11}$]6$^{11}$-2-furylmethylidenecyclohexanone-4-2-furylpyrrolidines (**3f)**

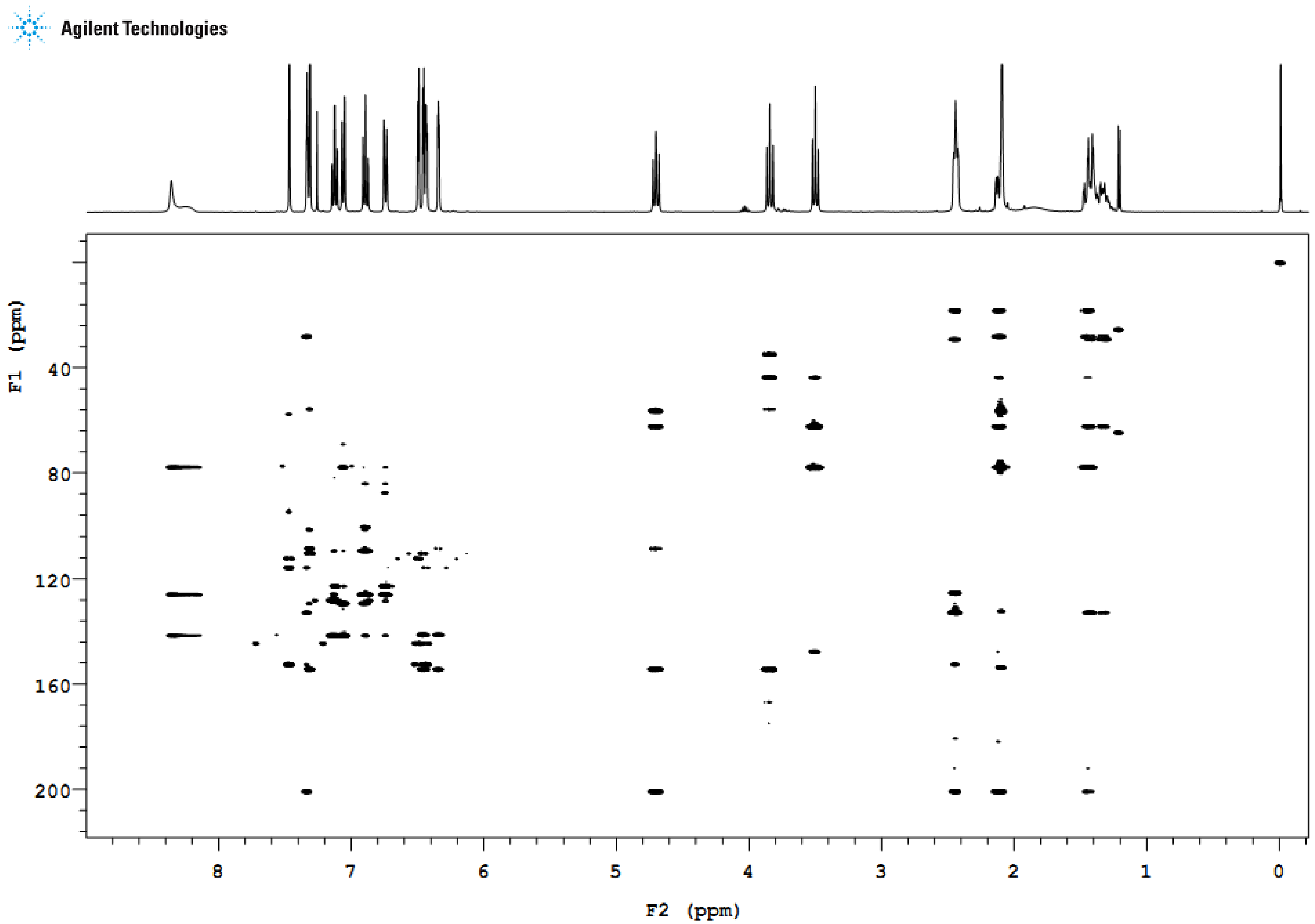


The $^{1}H$ $^{13}C$ HMBC spectrum 1-N-methyl-spiro[2.3$^{1}$]oxindole-spiro[3.2$^{11}$]6$^{11}$-2-furylmethylidenecyclohexanone-4-2-furylpyrrolidines (**3f)**

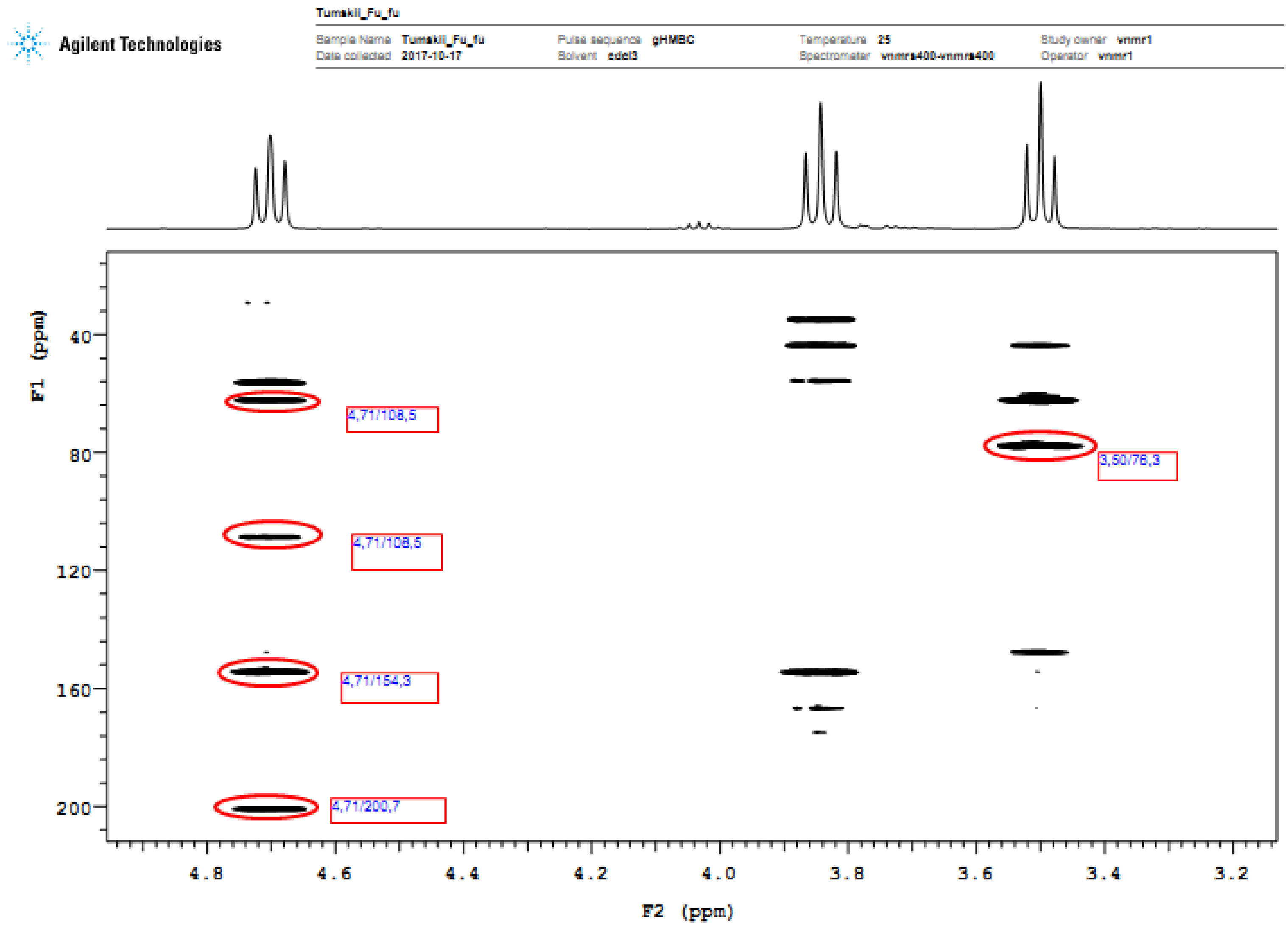


The detailed $^{1}H$ $^{13}C$ HMBC spectrum 1-N-methyl-spiro[2.3[1]]oxindole-spiro[3.2[11]]6[11]-2-furylmethylidenecyclohexanone-4-2-furylpyrrolidines (**3f)**

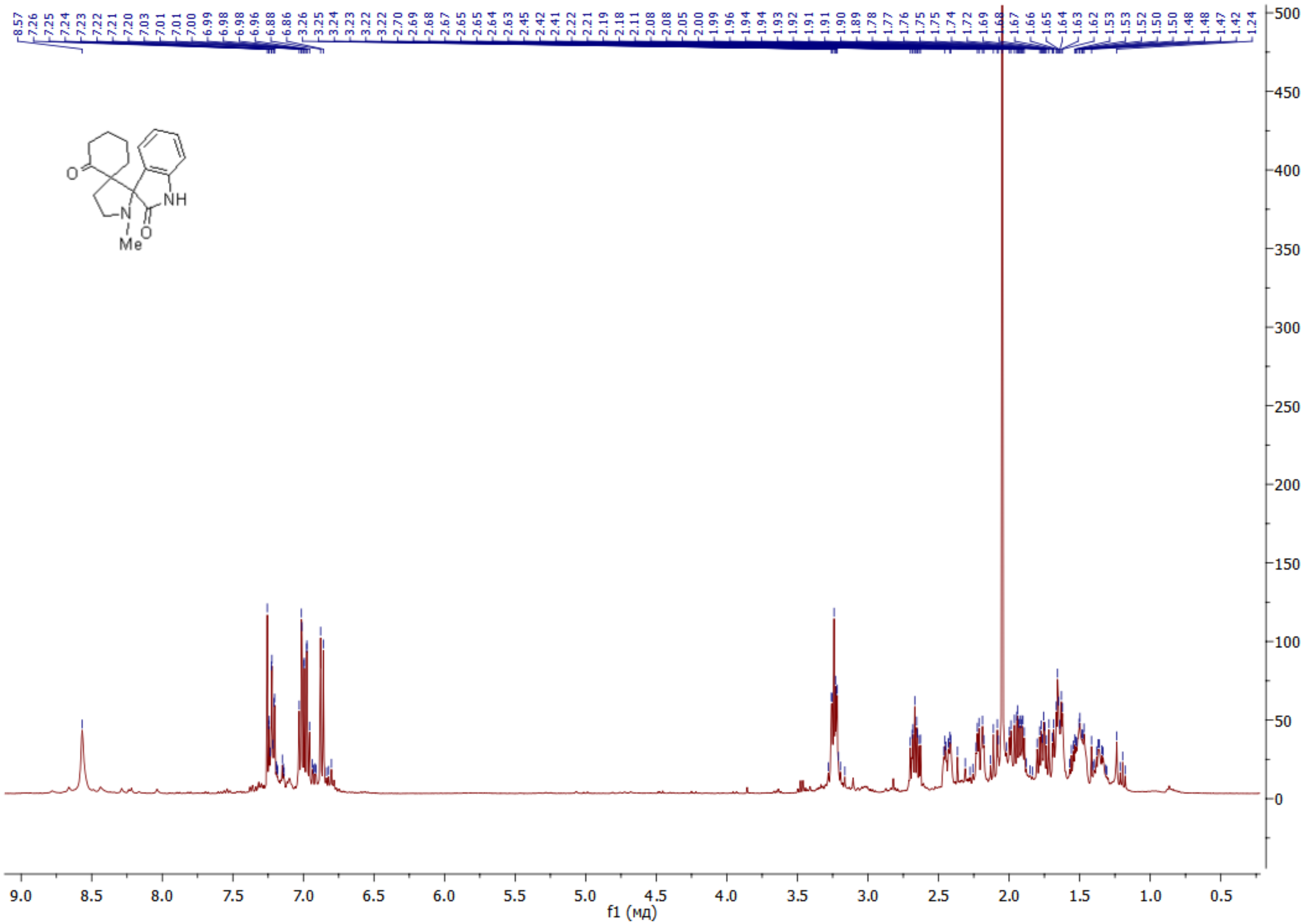


The $^{1}$H NMR 14-N-methyl-2,14-diazaspiro[4.0.5.3]tetradecan-3,4-benzo-1,7-dione **5**

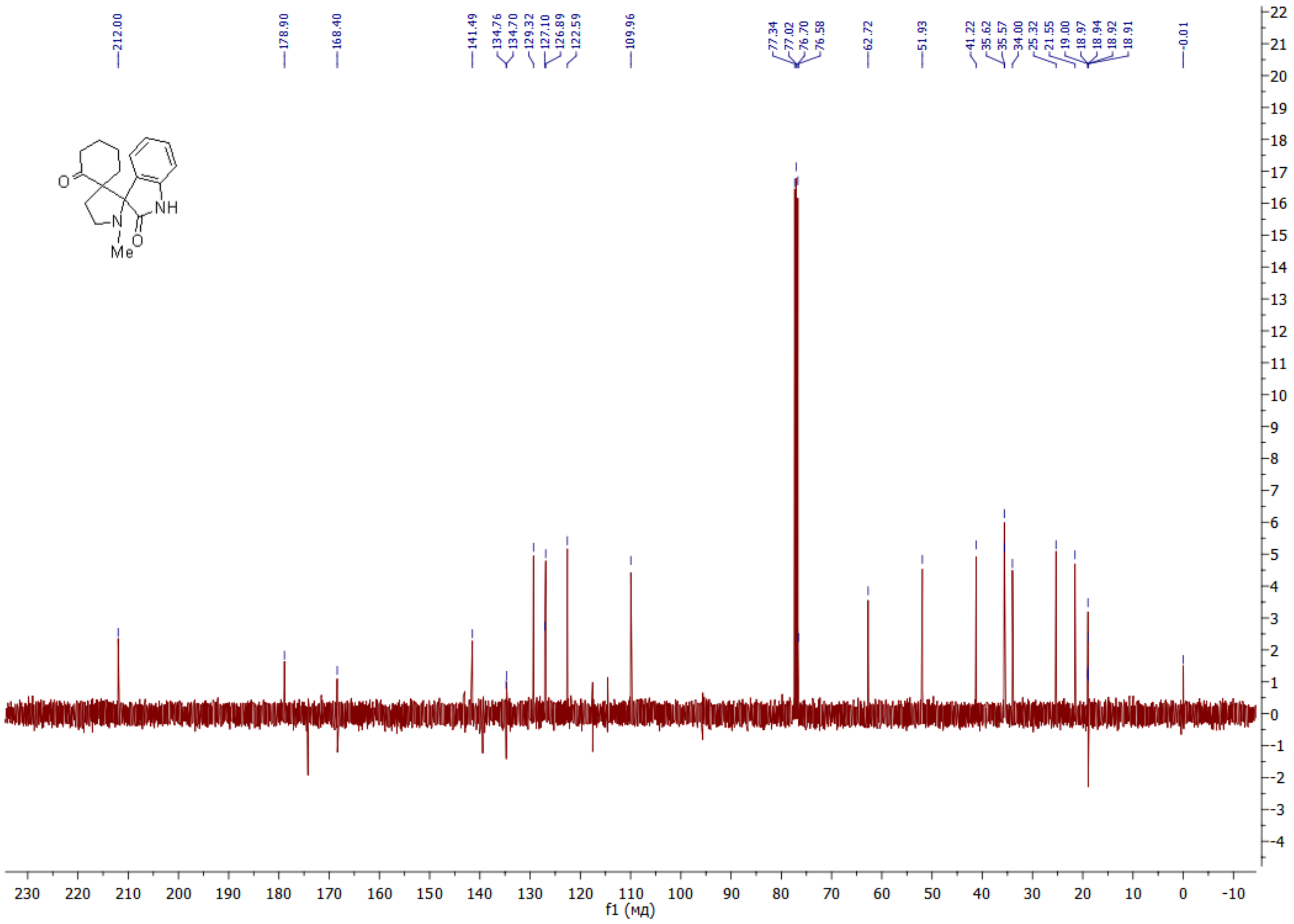


The $^{13}C$ NMR 14-N-methyl-2,14-diazaspiro[4.0.5.3]tetradecan-3,4-benzo-1,7-dione **5**

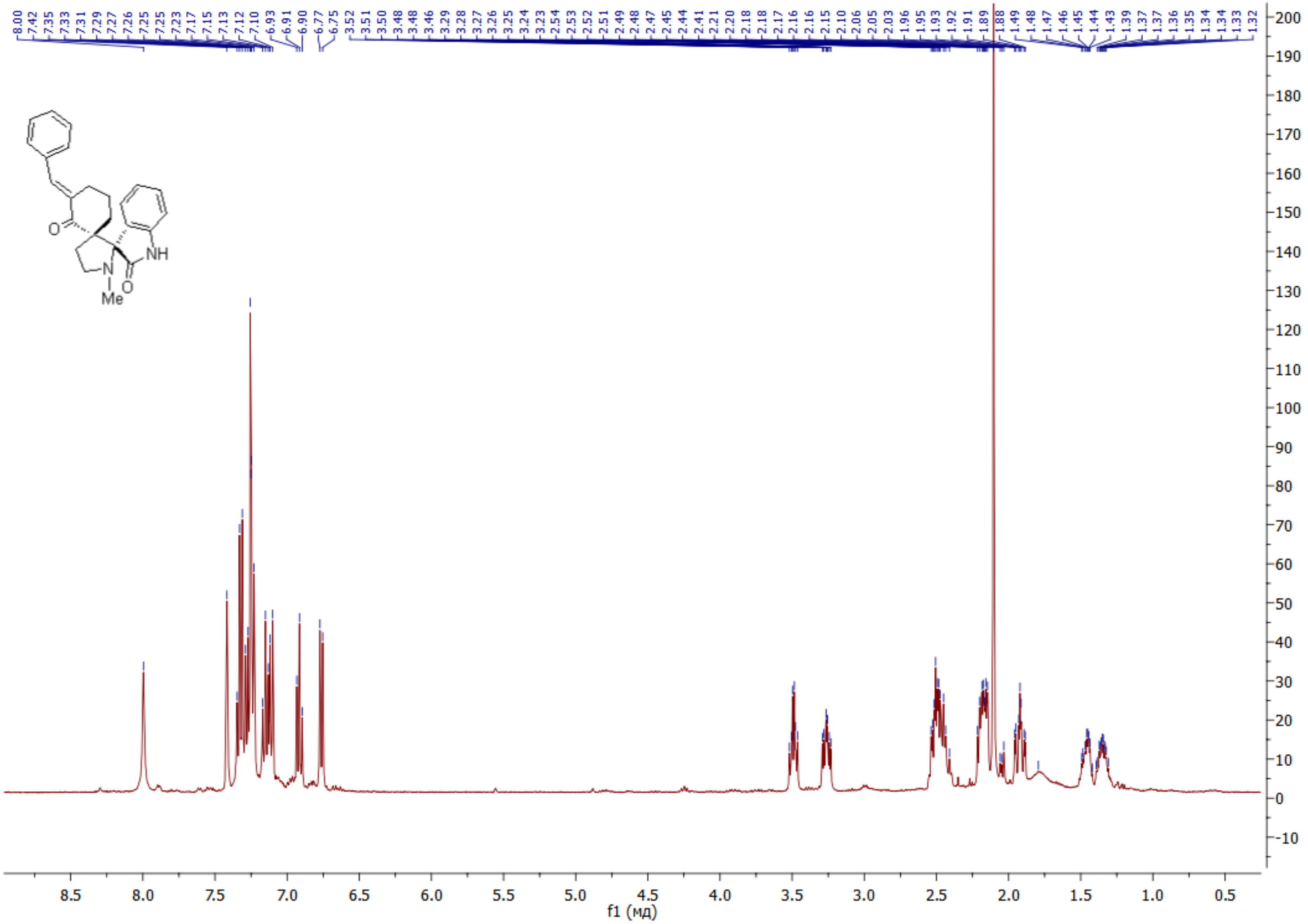


The $^{1}$H NMR 8-Phenylmethyliden-14-N-methyl-2,14-diazaspiro[4.0.5.3]tetradecan-3,4-benzo-1,7-dione **7**

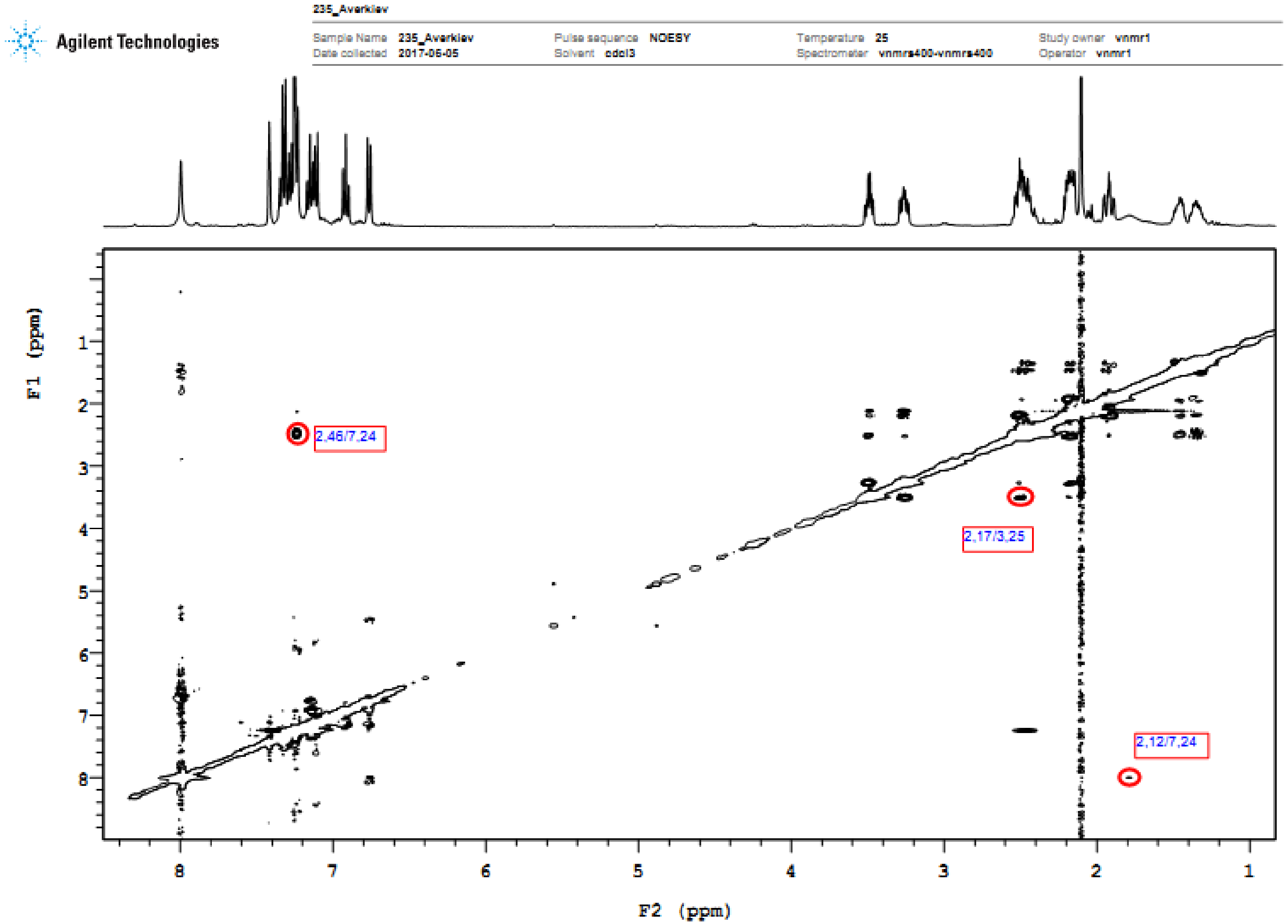


The NOESY spectrum 8-Phenylmethyliden-14-N-methyl-2,14-diazaspiro[4.0.5.3]tetradecan-3,4-benzo-1,7-dione **7**

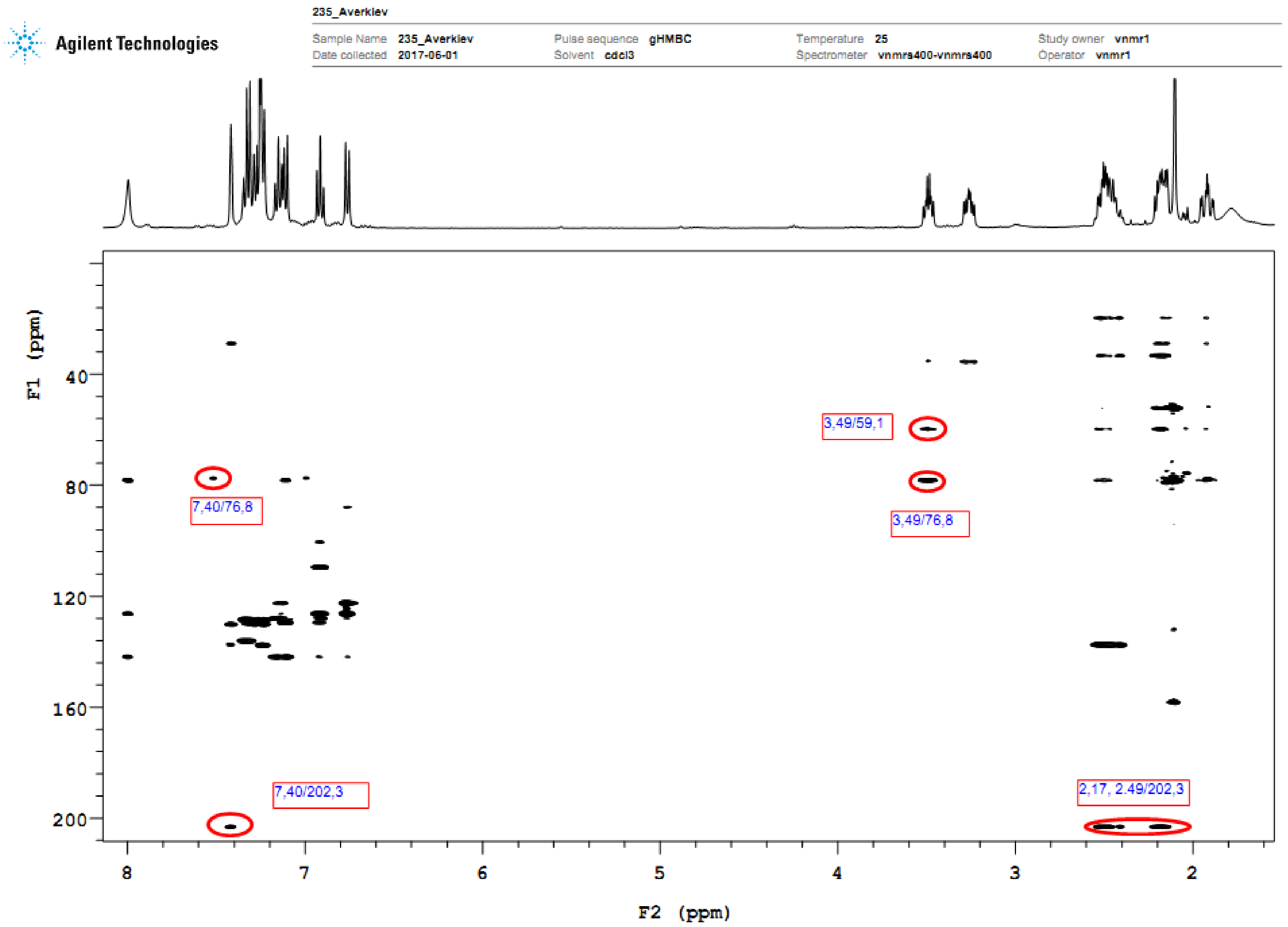


The $^1H^{13}C$ HMBC spectrum 8-Phenylmethyliden-14-N-methyl-2,14-diazaspiro[4.0.5.3]tetradecan-3,4-benzo-1,7-dione **7**

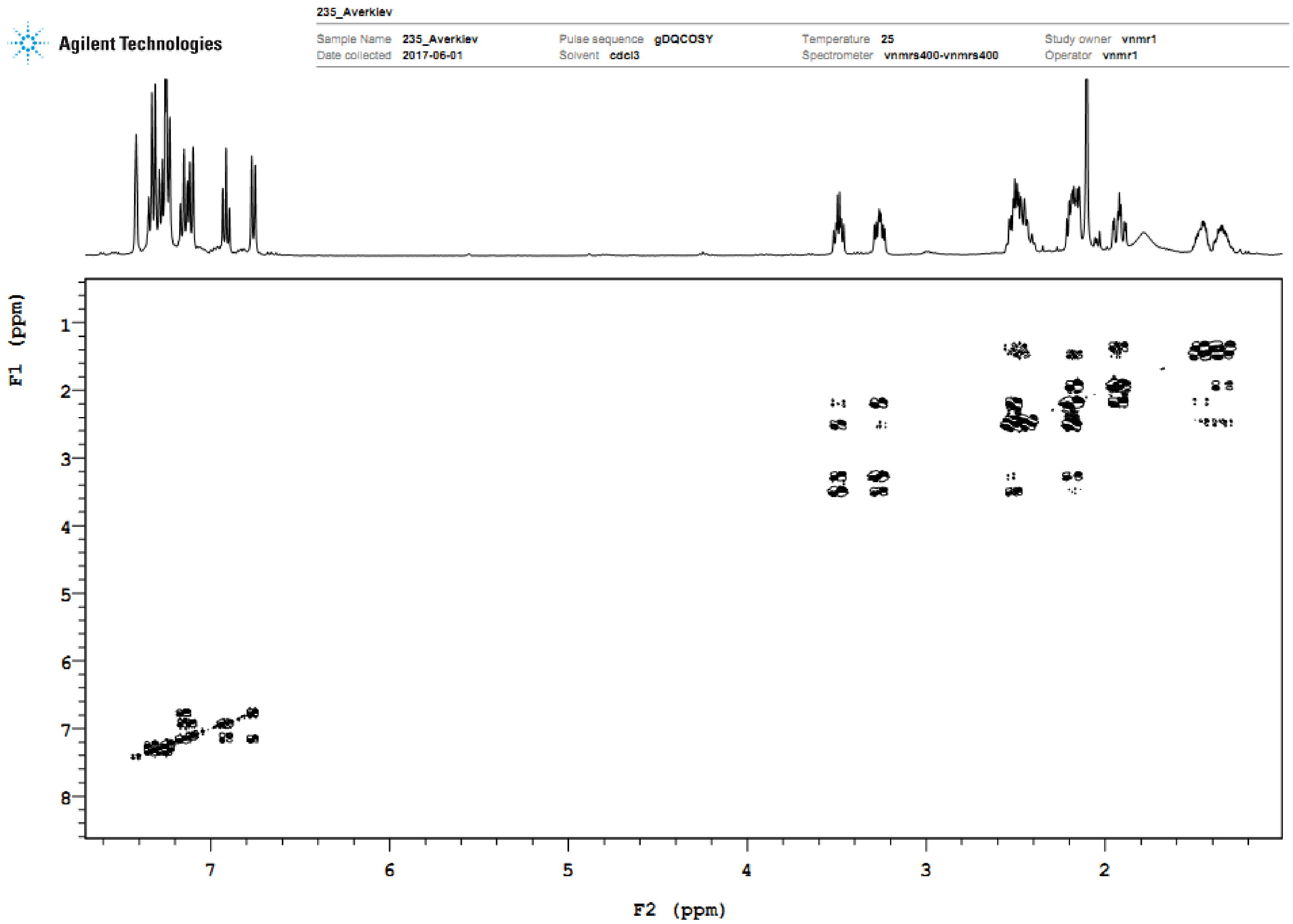


The dqCOSY spectrum 8-Phenylmethyliden-14-N-methyl-2,14-diazaspiro[4.0.5.3]tetradecan-3,4-benzo-1,7-dione **7**
